# Neuropeptide therapeutics to repress lateral septum neurons that disable sociability in an autism mouse model

**DOI:** 10.1016/j.xcrm.2024.101781

**Published:** 2024-10-17

**Authors:** Amélie M. Borie, Yann Dromard, Prabahan Chakraborty, Pierre Fontanaud, Emilie M. Andre, Amaury François, Pascal Colson, Françoise Muscatelli, Gilles Guillon, Michel G. Desarménien, Freddy Jeanneteau

**Affiliations:** 1 Institut de Génomique Fonctionnelle, Department of Neuroscience, Stress Hormones and Plasticity Unit, University of Montpellier, INSERM, CNRS, 34090 Montpellier, France; 2 Département de Maieutique, University of Montpellier, 34090 Montpellier, France; 3 Department of Anesthesiology and Critical Care Medicine, Arnaud de Villeneuve Academic Hospital, Montpellier 34090 Montpellier, France; 4 Institut de Neurobiologie de la Méditerranée, INSERM, University of Aix-Marseille, 13273 Marseille, France

**Keywords:** social, autism, Pader-Willi, Magel2, treatment, oxytocin, vasopressin, somatostatin, photometry, optogenetic

## Abstract

Confronting oxytocin and vasopressin deficits in autism spectrum disorders and rare syndromes brought promises and disappointments for the treatment of social disabilities. We searched downstream of oxytocin and vasopressin for targets alleviating social deficits in a mouse model of Prader-Willi syndrome and Schaaf-Yang syndrome, both associated with high prevalence of autism. We found a population of neurons in the lateral septum—activated on termination of social contacts—which oxytocin and vasopressin inhibit as per degree of peer affiliation. These are somatostatin neurons expressing oxytocin receptors coupled to GABA-B signaling, which are inhibited via GABA-A channels by vasopressin-excited GABA neurons. Loss of oxytocin or vasopressin signaling recapitulated the disease phenotype. By contrast, deactivation of somatostatin neurons or receptor signaling alleviated social deficits of disease models by increasing the duration of contacts with mates and strangers. These findings provide new insights into the treatment framework of social disabilities in neuropsychiatric disorders.

## Highlights

• Excess septal SST activity in the autism model terminates social contact
• Antagonism of septal SST alleviates social deficits in the autism model
• Septal SST cells depleted of OXT receptors in the autism model
• OXT signals via GABA-B and AVP via GABA-A to deactivate SST cells

## Graphical abstract

**Figure:**
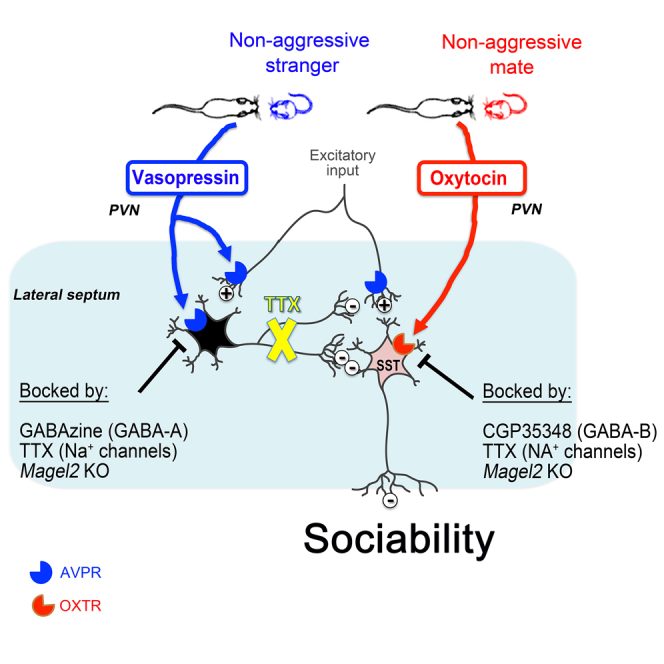

## Teaser

Social deficits cause poor clinical outcome in neuropsychiatric diseases, and treatments are lacking. Borie et al. identified a population of neurons activated on the termination of social contacts that fail to disengage in experimental autism. Suppressing the activity or signaling of these cells increased contact duration with mates and strangers.

## Introduction

Social deficits linked to autism spectrum disorders (ASDs) are reported in human carriers of *de novo* point mutations in the *MAGEL2* gene, who develop Schaaf-Yang syndrome,^1^ and are also reported in patients with chromosomal alterations including *MAGEL2*, who develop Prader-Willi syndrome.^2^ More than 75% of patients with Schaaf-Yang syndrome and 25% with Prader-Willi syndrome have ASD.^3^^,^^4^ Despite the genetic heterogeneity between patients, the *MAGEL2* gene has been a central focus of studies for disease mechanisms and innovative treatments.^5^

Developmentally, *MAGEL2* is expressed *in utero* onward, mostly in GABA neurons and peptidergic neurons of the septum and hypothalamus (e.g., vasopressin [AVP] and oxytocin [OXT]), where it acts as a co-factor of E3-ubiquitin ligases for protein clients linked to vesicular recycling,^6^ and lesser in excitatory neurons.^7^ Loss of MAGEL2 in patient-derived cells causes a paucity of secretory granules and reduces the production of neuropeptides like AVP, OXT, and somatostatin (SST).^8^

Consistent with the human pathology, transgenic mice with the gene *Magel2* knocked out (KO) express lower quantity of mature hypothalamic peptides secreted in the brain, which is linked to early-onset deficits in feeding, cognitive, and social behaviors.^5^^,^^9^ Altered social behaviors in *Magel2*KO mice have been attributed in part to deficits of preference for novelty^10^ due to a paucity of hypothalamic peptides signaling in the forebrain.^11^ Memory deficits of the *Magel2*KO model correspond with forebrain activity dysfunctions recorded in the theta band that is specifically triggered by social stimuli but not with objects.^11^

To confront some of these deficits, we previously treated *Magel2*KO mice with OXT or AVP, which independently improved forebrain theta band activity and feeding, cognitive, and social behaviors.^11^^,^^12^ Similar treatments via the nasal route in patients with ASD also improved social communication.^13^^,^^14^ In babies and young children with Prader-Willi syndrome, treatment with intranasal OXT improved feeding and social engagement.^15^^,^^16^ However, replicate studies in subjects with ASD and Prader-Willi are *not* consistent.^17^^,^^18^ Possible explanations for the conflicting results include the variations in therapeutic windows, the genetic heterogeneity between patients, and the role of OXT and AVP on both pro-social and agonistic behaviors.^19^ Besides, polygenic association studies already linked ASD with polymorphisms in both human OXT receptor (OXTR) and AVP receptor 1A (AVPR1A),^20^ suggesting that dysfunctions in both systems interact.

We propose that counterproductive effects of treatments reflect dysfunctional interaction of AVP and OXT, which need to be dissected. Firstly, *when* and *where* AVP and OXT are released in the brain depend on experiential context. For instance, acquiring social safety after social trauma engages OXT from the supraoptic nucleus (SON) but not from the paraventricular nucleus (PVN).^21^ Secondly, the response to AVP and OXT depends on discrete “functional units” wherein interacting neurons with non-overlapping expression of cognate receptors work in tandem as a unit to control opposing behaviors.^22^ For instance, we characterized SST neurons harboring OXTR as one arm of such functional units while the other arm is operated by neurons containing AVPRs.^23^ This raises the question whether the source of neuropeptide release could trigger opposing responses via such functional units. Recently, we validated this possibility in fear-related behaviors,^21^^,^^23^ but it remains largely unknown in socially non-aversive context, which are also impaired in ASD.

To address this gap in knowledge, we explored how the convergence of OXT and AVP systems on the modulation of SST cells modulates the sociability of *Magel2*KO mice toward non-threatening strangers. We focused on the lateral septum (LS) where functional interplay is expected due to the high density of SST cells and OXT and AVP fibers and receptors.^23^ Previously, the down-regulation of either OXTR^24^ or AVPR1A^25^ in the LS of healthy rodents caused social memory impairment; and prairie voles co-infused with OXT plus AVPR1A antagonist in the LS preferred huddling with cage-mates over a stranger contrary to prairie voles co-injected with OXT plus OXTR antagonist that showed no preference.^26^ To date, the role of septal SST cells and their modulation by OXT and AVP has been largely ignored in the pathological framework despite the abundant expression of *MAGEL2* in the human septal nuclei (Allen Brain Atlas).

There is an undisputed consensus that brain SST levels go down in neurodegenerative diseases.^27^ Reduced cortical SST levels have been reported in schizophrenia, epilepsy, and mood disorders as well but the septum has been overlooked.^27^ SST neurons are short-range GABAergic interneurons exhibiting regular spiking activity that contribute to theta rhythms underlying cognitive functions.^28^ Molecular diversity specifies a variety of SST neuron subtypes associated with distinct functions, some even making long-range projections.^29^ The role of SST neuron subtype modulated by OXT and AVP remains largely unexplored in the context of ASD.

Here, we found that AVP signals via post-synaptic GABA-A channels and OXT via GABA-B receptors to inhibit SST cells in the LS. Loss of OXT and AVP support in the LS locks local circuitry into a hyper-somatostatinergic state that promotes the termination of social contacts. Blockade of SST signaling offset the loss of OXT and AVP support in the LS of *Magel2*KO mice and increased contacts with non-threatening strangers. These results have far-reaching implications for the treatment of social deficits often associated with poor clinical outcomes in numerous neuropsychiatric and neurodevelopmental diseases.

## Results

### Excessive SST cell activity on the termination of social contacts in the LS of *Magel2*KO mice

**Figure 1:**
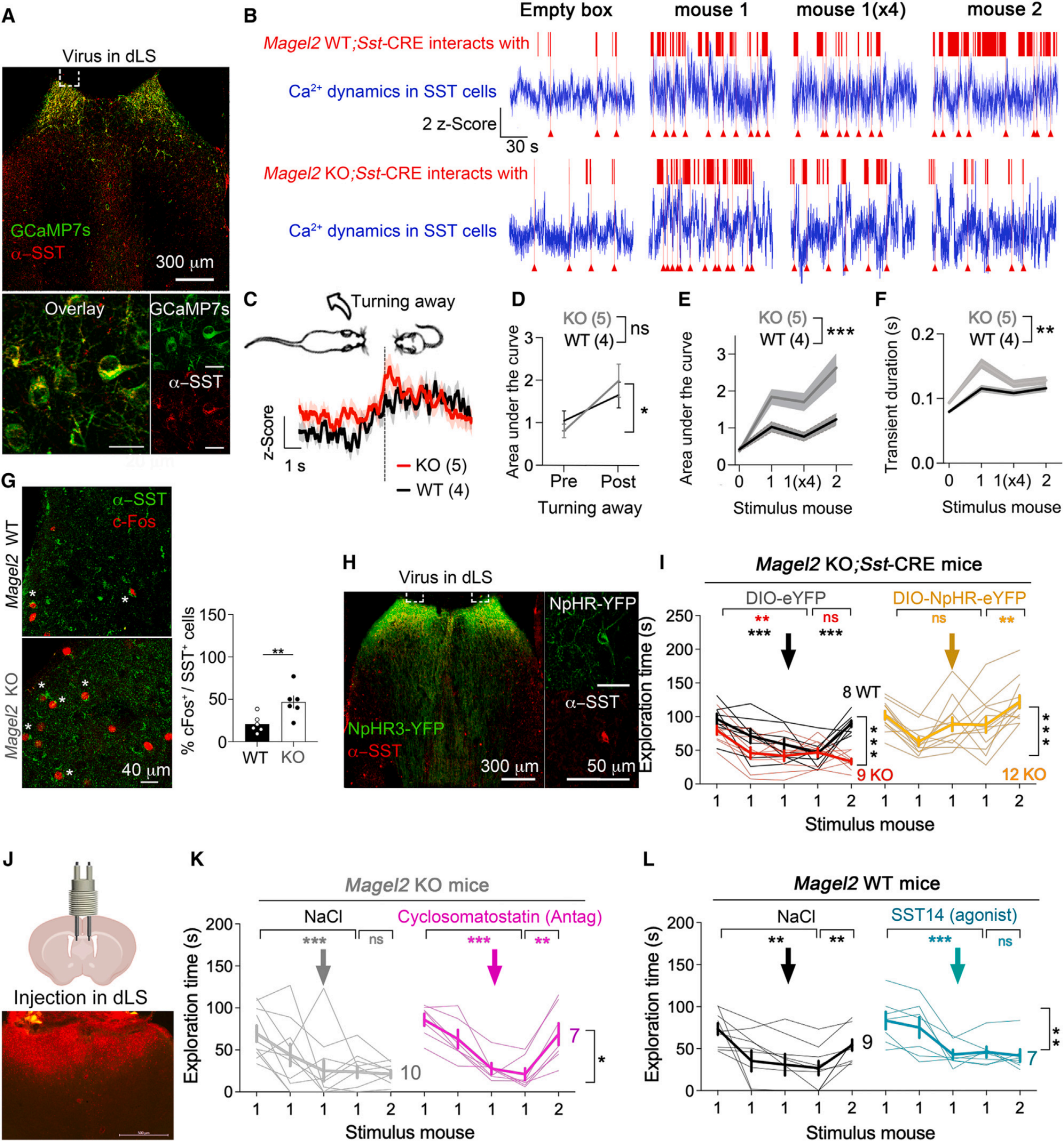
Deactivation of SST signaling in dLS corrects social deficits of *Magel2*KO mice (A) Expression of CRE-dependent GCaMP7s in dLS of *Magel2*KO crossed with *Sst*-CRE mice. Scale, 20 μm. (B) Photometry traces during exploration of the empty stimulus box, then once with mouse 1, up to 4 times (×4), and then once with the unknown mouse 2. Arrowheads point at turning away events. See Figure S1A for details of the behavioral task. (C) Ca^2+^ dynamics when subjects turn away from the stimulus mouse (see Video S1). Means ± SEM, *n* = 22 WT events, 29 KO. Two-way ANOVA: effect of genotype *F*_(1,255317)_ = 1,393 *p* < 0.0001, effect of time *F*_(4822, 255317)_ = 2.9 *p* < 0.0001. Video S1. Changes of Ca^2+^ dynamics in SST neurons of dLS when turning away from the stimulus mouse, related to Figure 1(A) Recombinant expression of tracer.(B) Photometry traces.(C) Ca^2+^ dynamics during behavior.(D) Ca^2+^ dynamics locked to one behavior.(E) Area under the curve.(F) Peak duration.(G) Maker of neuronal activation in dLS.(H) Recombinant expression of opsin.(I) Optogenetic inhibition on behavior.(J) Bilateral cannulas for drug infusion in dLS.(K) Pharmacological antagonism of somatostatin receptors. (D) Change of Ca^2+^ dynamics before and after turning away events in *n* = 4 WT and 5 KO mice. Two-way ANOVA pre vs. post event *F*_(1,48)_ = 11.4 *p* = 0.001 post hoc Sidak test *p* = 0.008. Cohen’s *d* effect size bigger in KO (*d* = 0.75) than WT (*d* = 0.43). See Figures S1B–S1G for controls. (E) Area under the curve during each stimulus trial (*n* = 41 empty, 70 mouse 1, 61 mouse 1 (×4), and 68 mouse 2 for 4 WT mice and *N* = 37, 66, 41, and 51 for 5 KO). Two-way ANOVA: trial × genotype *F*_(3,433)_ = 3.6 *p* = 0.01 post hoc Sidak test. Cohen’s *d* effect size bigger in KO (*d* = 1.05) than WT (*d* = 0.64). (F) Peak duration during each stimulus trial (*N* = 331 empty, 276 mouse 1, 275 mouse 1 (×4), and 240 mouse 2 for 4 WT mice and *N* = 341, 218, 264, and 214 for 5 KO). Two-way ANOVA: trial × genotype *F*_(3,1415)_ = 2.8 *p* = 0.03 post hoc Sidak test. (G) Fos-positive SST cells in dLS 1 h after all stimulus trials (Means ± SEM, *n* = 6 mice/group). Mann-Whitney test *p* = 0.0022 with large effect size Cohen’s *d* = 1.72. (H) Expression of CRE-dependent NpHR3-eYFP in dLS of *Magel2*KO;*Sst*-CRE mice. (I) Social deficits of *Magel2*KO;*Sst*-CRE mice corrected by optogenetic inhibition of SST cells in dLS. Two-way ANOVA: effect of genotype *F*_(1,15)_ = 9.9 *p* = 0.006, interaction of time × genotype *F*_(4,60)_ = 5.8 *p* = 0.0005 with large effect size during discrimination Cohen’s *d* = 4.2, effect of NpHR3 versus YFP in the KO group *F*_(1,19)_ = 25.9 *p* < 0.0001; interaction time × NpHR3 in the KO group *F*_(4,76)_ = 8.6 *p* < 0.0001, post hoc analysis with Sidak test and a large effect size during discrimination Cohen’s *d* = 3.36. (J) Diffusion area of a fluorescent tracer injected in dLS with bilateral cannulas. Scale, 500 μm. (K) Social deficits of *Magel2*KO mice corrected by intraseptal injection of 2 μg/side cyclosomatostatin (SSTR pan-antagonist). Means ± SEM, *n* mice as indicated. Two-way ANOVA: time × SSTR antagonism *F*_(4,57)_ = 2.6 *p* = 0.04, post hoc analysis with Dunnett test and a large effect size during discrimination Cohen’s *d* = 1.72. (L) Intraseptal injection of 1 ng/side SST14 (SSTR agonist) caused social deficits in *Magel2*WT mice. Means ± SEM, *n* mice as indicated. Two-way ANOVA: time × SST *F*_(4,50)_ = 3.6 *p* = 0.0106, post hoc analysis with Dunnett test and a large effect size during discrimination Cohen’s *d* = 1.6.

To investigate SST neuron activity in a socially non-aversive context, we recorded the dynamics of the Ca^2+^ indicator GCaMP7s during the exploration of same-sex juveniles as stimulus mice to avoid aggression and socio-sexual bias.^30^ The task consisted of 4 successive presentations with a juvenile in a stimulus box for 5 min with 20-min intervals to become familiar, followed by the presentation of a new estranged juvenile to unveil *Magel2*-related social deficits of preference for novelty (Figure S1A). A CRE-dependent virus was injected in the LS of double transgenic mice *Magel2*KO;*Sst*-CRE to express GCaMP7s in SST cells with 88% recombination rate (Figure 1A). The photometry fiber was implanted in dorsal LS (dLS) to record fluorescence dynamics during the presentation of a stimulus box without or with a known juvenile followed by a stranger (Figure 1B). We observed anti-correlated fluorescence dynamics with exploration bouts across trials (Figure 1B). In particular, changes of fluorescence dynamics corresponded with the termination of social contact when subjects turned away from stimulus mice (ANOVA wild type [WT] vs. KO comparison *p* < 0.0001, Figure 1C). This response is specific because there was no change of fluorescence dynamics related to either turning away from an object, turning forward to contact with an object, or turning forward to contact a stimulus mouse (Figures S1B–S1G). Although changes of Ca^2+^ dynamics occurred in both genotypes (ANOVA pre vs. post, *p* = 0.001 not different between WT and KO *p* = 0.7, Figure 1D), their amplitude measured by the area under the curve (ANOVA *p* = 0.01, Figure 1E) and duration measured by the width at the base of the curve (ANOVA *p* = 0.03, Figure 1F) increased in *Magel2*KO mice compared to WT controls. Mice were sacrificed 1 h after social stimulation for histological analysis. In agreement with the increase of Ca^2+^ dynamics, we counted more Fos-activated SST cells in the LS of *Magel2*KO mice than in WT controls (Mann-Whitney test *p* = 0.002, Figure 1G). These data suggest that an excessive engagement of SST cells might lead to premature termination of social contacts.

### Deactivation of SST signaling in the LS corrects the social deficits of *Magel2*KO mice

To interrogate the role of SST cells specifically, we virally introduced the CRE-dependent halorhodopsin NpHR3-eYFP (or eYFP as control) in the LS of double transgenic mice *Magel2*KO;*Sst*-CRE with 77% efficiency and implanted bilateral optic fibers atop (Figure 1H). Continuous yellow light was applied after familiarization with the 1^st^ stimulus juvenile to assess the impact on contacts with a stranger because *Magel2*KO mice exhibit social deficits in this test (WT vs. KO with ANOVA *p* < 0.0001, Figure 1I). We found that optogenetic silencing of SST cells in *Magel2*KO mice increased contacts with a stranger compared to eYFP KO controls (ANOVA *p* < 0.0001, Figure 1I). To further distinguish between local and long-distance SST signaling possibly engaged in social deficits, we infused a pan-antagonist of SST receptors (SSTRs) through bilateral cannulas in dLS where the diffusion area was validated with a fluorescent tracer (Figure 1J). There are 4 SSTR subtypes expressed in the LS.^31^ Infusion of the non-selective SSTR antagonist cyclosomatostatin during social familiarization subsequently increased contacts with a stranger, compared to NaCl-injected KO controls (ANOVA *p* = 0.04, Figure 1K). On the contrary, intra-septal infusion of the agonist SST14 in *Magel2*WT mice decreased the duration of contacts with a stranger compared to NaCl-injected WT controls (ANOVA *p* = 0.01, Figure 1L). These results indicate that a disengagement of local SST signaling led to longer contacts with a stranger.

### *Magel2* KO impairs signaling of OXT via GABA-B and AVP via GABA-A in SST cells of the LS

**Figure 2:**
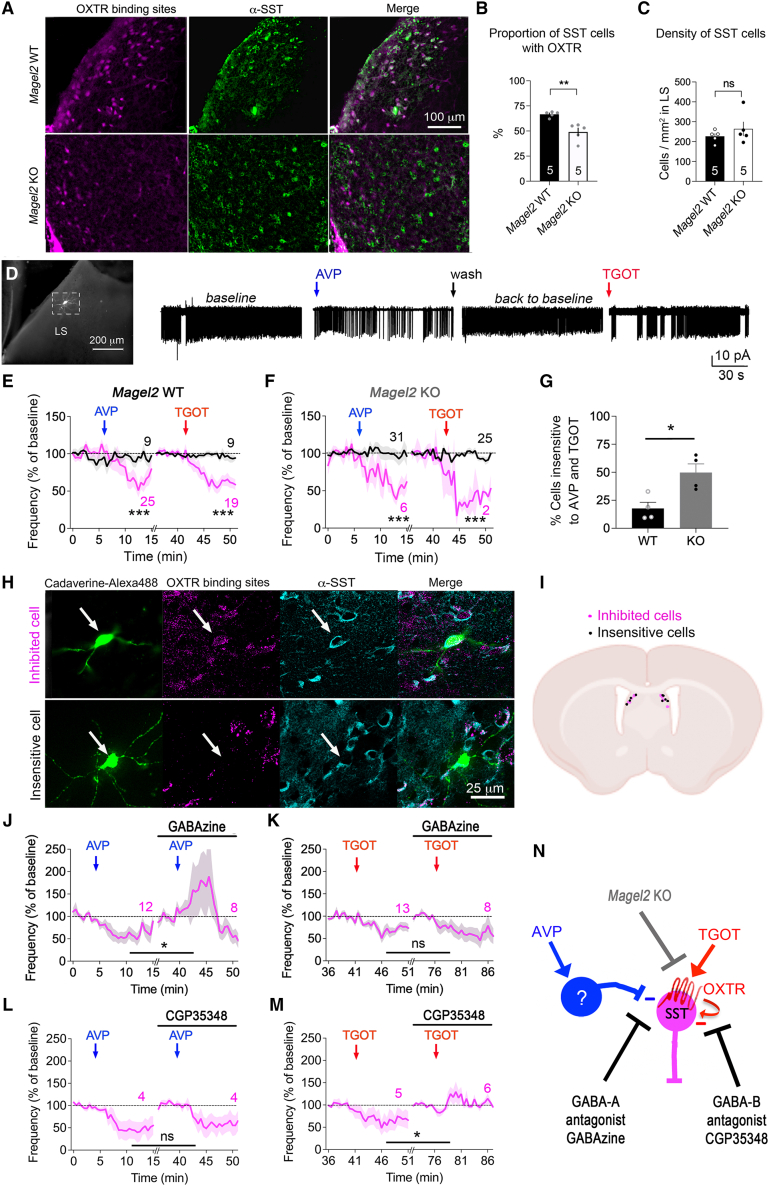
OXT and AVP inhibit septal SST cells less efficiently in *Magel2*KO mice (A) Binding of 150 nM d[Lys(Alexa 647)^8^]VP marked OXTR mostly in SST cells of LS labeled post-fixation with SST antibodies. (B) Proportion of OXTR+SST cells reduced in dLS of *Magel2*KO mice compared to WT (means ± SEM, *n* = 5 mice/group). Mann-Whitney test *p* = 0.007 with a large effect size Cohen’s *d* = 2.7. (C) Density of SST cells unchanged by *Magel2* deficiency (means ± SEM, *n* = 5 mice/group). Mann-Whitney test *p* = 0.6, Cohen’s *d* = 0.6. (D) Single cell recording of action potentials upon perfusion with 1 μM AVP then 0.1 μM TGOT for 2 min with at least 20 min wash inter-trial to get back to baseline activity, and finally filled the Alexa 488-cadaverine tracer. (E) Effect of AVP and TGOT on spike frequency in acute slices of *Magel2*WT mice. Three-way ANOVA: effect of stimulations *F*_(30,1709)_ = 4.07 *p* < 0.0001 distinguished the responding cells (purple line) from the insensitive (black line): *F*_(1,58)_ = 5.2 *p* = 0.026; effect of inhibition by AVP or TGOT is not different *F*_(1,58)_ = 0.05 *p* = 0.8 post hoc Tukey’s test. See Figures S2F and S2G for the sequential effect. (F) Effect of AVP and TGOT on spike frequency in acute slices of *Magel2*KO mice. Three-way ANOVA: effect of stimulations *F*_(30,1752)_ = 5.6 *p* < 0.0001 distinguished the responding cells (purple line) from the insensitive (black line): *F*_(1,60)_ = 23.4 *p* < 0.0001; no effect of genotype between responding cells *F*_(1,60)_ = 1.9 *p* = 0.17 post hoc Tukey’s test. See Figures S2A–S2E for controls. (G) Three times more cells insensitive to AVP and TGOT in slices of *Magel2*KO mice than WT controls. Mean ± SEM, *n* = 9 cells WT and 31 KO, Mann-Whitney test *p* = 0.028 with a large effect size Cohen’s *d* = 2.38. (H) Binding of 150 nM d[Lys(Alexa 647)^8^]VP on live slices marked with the cadaverine-Alexa 488 filler, and SST antibodies post-fixation. Binding to d[Lys(Alexa 647)^8^]VP discriminated between TGOT-inhibited and insensitive cells. (I) Localization of TGOT-inhibited and insensitive SST cells in dLS. (J) GABA-A antagonist (GABAzine 0.3 μM) blocked the effect of AVP on spike frequency. Two-way ANOVA: effect of AVP *F*_(30,330)_ = 2.7 *p* < 0.0001, GABAzine *F*_(1,11)_ = 4.7 *p* = 0.05, and AVP × GABAzine *F*_(30,169)_ = 4.05 *p* < 0.0001 post hoc Dunnett test, *n* cells as indicated. (K) Effect of TGOT not blocked by GABAzine. Two-way ANOVA: effect of TGOT *F*_(30,390)_ = 7.44 *p* < 0.0001, GABAzine *F*_(1,13)_ = 0.32 *p* = 0.5, and TGOT × GABAzine *F*_(30,164)_ = 0.87 *p* = 0.6 post hoc Dunnett test, *n* cells as indicated. (L) Effect of AVP not blocked by GABA-B antagonist (CGP35348 100 μM, 5 min before). Two-way ANOVA: effect of AVP *F*_(30,90)_ = 5 *p* < 0.0001, interaction with CGP *F*_(30,90)_ = 0.3 *p* = 0.9 post hoc Dunnett test, *N* cells as indicated. (M) CGP35348 blocked the effect of TGOT on spike frequency. Two-way ANOVA: effect of TGOT *F*_(30,150)_ = 1.3 *p* = 0.1, interaction with CGP *F*_(30,119)_ = 3.1 *p* < 0.0001 post hoc Dunnett test, *N* cells as indicated. (N) Proposed model. TGOT signals via GABA-B while AVP signals via GABA-A transmission. See Figure S3 for the effect of TTX on synaptic events and its modulation by OXT and AVP.

Given that a majority of SST neurons in the LS express OXTR, we examined the effect of genotype using the fluorescent OXTR ligand d[Lys(Alexa Fluor 647)8]VP previously characterized.^23^ It is best used on live tissue and was further counterstained with SST antibodies (Figure 2A). We found an effect of genotype on the proportion of SST cells positive with OXTR-binding sites (WT vs. KO with Mann-Whitney *p* = 0.007, Figure 2B). The lower number of OXTR-binding sites in *Magel2*KO mice did not merely account for a reduction in SST cell density compared to WT controls (Mann-Whitney *p* = 0.6, Figure 2C). To characterize functional implications, we patched cells in acute slices and recorded the frequency of action potentials before and after stimulation for 2 min with 1 μM AVP, followed by 1 μM TGOT (OXTR agonist) (Figure 2D). Cells lost ∼50% of basal activity following stimulations (Figure 2E), and it was reversible (Figure 2D) (responding vs. insensitive cells ANOVA *p* < 0.0001). In *Magel2*KO slices, the basal firing rate was normal (Figures S2A–S2E), and the response to AVP and TGOT did not significantly differ from WT controls (ANOVA *p* = 0.4, Figure 2F). However, there was a significant drop in the number of KO cells responding to both peptides compared to WT controls, thereby enhancing the proportion of insensitive cells in mutants (Mann-Whitney *p* = 0.028, Figure 2G). To address the possibility that insensitive KO cells lack OXTR, we labeled live slices with d[Lys(Alexa Fluor 647)8]VP before infusing the fluorescent filler cadaverine and counterstaining with SST antibodies. Cells inhibited by TGOT were double positive for OXTR-binding sites and SST immunolabeling (Figure 2H). Such SST cells insensitive to TGOT lacked OXTR-binding sites and were intermingled with the SST-responding cells in the LS, making it impossible to distinguish by anatomy (Figure 2I).

Given that OXT and AVP affect inhibitory responses, we next investigated a role of GABA receptors. The GABA-A receptor antagonist GABAzine blocked responses evoked by AVP (ANOVA *p* < 0.0001, Figure 2J) but not by TGOT (ANOVA *p* = 0.9, Figure 2K). Conversely, the GABA-B receptor antagonist CGP35348 blocked responses evoked by TGOT (ANOVA *p* < 0.0001, Figure 2M) but not by AVP (ANOVA *p* = 0.9, Figure 2L). Interestingly, TGOT-mediated inhibition of SST cells depended on prior application of AVP (Figures S2F and S2G), suggesting it may rely on local spontaneous network activity. Indeed, the Na^+^ channel blocker tetrodotoxin (TTX) prevented synaptic events evoked by AVP and TGOT (Figure S3). We concluded that OXTR signaled via GABA-B in SST cells, while AVP required post-synaptic GABA-A transmission, with *Magel2* inactivation decreasing OXTR signaling in these cells (Figure 2N).

### AVPR and OXTR deactivate SST cells as per degree of peer affiliation, a requirement for the treatment of *Magel2*KO phenotype

**Figure 3:**
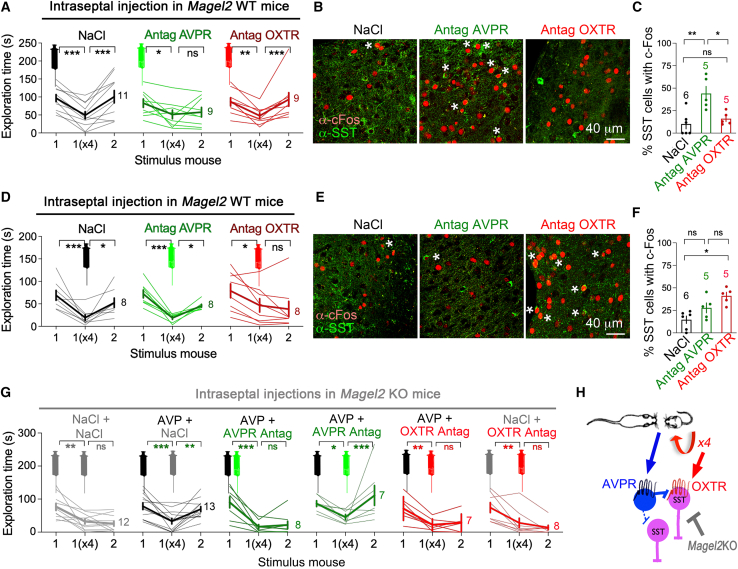
AVPR and OXTR inhibit septal SST cells as per degree of peer affiliation, and both are required to alleviate the *Magel2*KO phenotype (A) Intraseptal injection during social novelty of 10 nM Manning compound (AVPR antagonist) or 10 nM atosiban (OXTR antagonist)^36^ in *Magel2*WT mice (means ± SEM, *n* as indicated). Two-way ANOVA: interaction of Manning compound × stimulus mice *F*_(2,36)_ = 4.85 *p* = 0.013 unlike atosiban × stimulus mice *F*_(2,36)_ = 0.18, *p* = 0.8 post hoc with Dunnett test and Cohen’s *d* effect size bigger with Manning compound (*d* = 0.85) than atosiban (*d* = 0.13). (B) Fos-expressing SST cells in LS 0.5 h after the last stimulus trial. (C) Effect of antagonists on the proportion of Fos-activated SST cells in *Magel2*WT (means ± SEM, *n* as indicated). One-way ANOVA *F*_(2,12)_ = 14.4 *p* = 0.007, post hoc Dunnett test NaCl vs. Manning *p* = 0.0005 and Cohen’s *d* = 2.19, NaCl vs. atosiban *p* = 0.3 and Cohen’s *d* = 0.5. (D) Intraseptal infusion during social habituation of 10 nM Manning compound or 10 nM atosiban. Time of social contact (means ± SEM) in *Magel2*WT mice (*n* as indicated). Two-way ANOVA: interaction of atosiban × stimulus mice *F*_(2,28)_ = 3 *p* = 0.06, Manning compound × stimulus mice *F*_(2,28)_ = 0.24 *p* = 0.7, effect of stimulus mice *F*_(2,28)_ = 26.9 *p* < 0.0001, post hoc analysis with Dunnett test. (E) Fos-expressing SST cells in LS 0.5 h after the last stimulus trial. (F) Effect of antagonists on the proportion of Fos-activated SST cells in *Magel2*WT mice. Means ± SEM, *n* as indicated. One-way ANOVA *F*_(2,13)_ = 8.7 *p* = 0.0039, post hoc Dunnett test NaCl vs. Manning *p* = 0.1 and Cohen’s *d* = 1, NaCl vs. atosiban *p* = 0.002 and Cohen’s *d* = 2.7. (G) Intraseptal infusion of 10 pg/side AVP during novelty and 10 nM Manning compound or 10 nM atosiban during habituation in *Magel2*KO mice. NaCl was used as control (means ± SEM, *n* as indicated). Two-way ANOVA: antagonists × stimulus mice *F*_(10,98)_ = 3.38 *p* = 0.0008, effect of antagonists *F*_(5,49)_ = 3.2 *p* = 0.01 post hoc Dunnett test: effect of AVP vs. NaCl during novelty (*p* = 0.03 and Cohen’s *d* = 1.3), AVP + Manning compound during novelty (*p* = 0.012 and Cohen’s *d* = 1.58), AVP + atosiban during habituation (*p* = 0.002 and Cohen’s *d* = 1.34). (H) Proposed model. AVP facilitates GABA-A transmission onto OXTR+ SST cells and perhaps also onto OXTR− cells.

To determine the behavioral relevance of this model, we injected antagonists during social novelty or familiarization specifically in the LS where previous studies showed AVP concentration to surge after one social encounter,^32^ whereas it is the repetition of this contact that released OXT.^33^ We predicted that *in vivo*, effects of these neuropeptides might scale with the degree of peer affiliation, variably affecting the integration of familiar and stranger cues. To test this, bilateral cannulas were implanted in dLS to infuse 10 nM antagonists of AVPRs (Manning compound) or OXTR (atosiban) because they are selective at this dose in the mouse brain.^34^^,^^35^ We found that in WT mice, blocking AVPRs during novelty reduced later contacts with a stranger compared to NaCl-injected controls, while blocking OXTR at novelty had no effect (one-way ANOVA NaCl vs. antagonists *p* = 0.03, Figure 3A). Mice were sacrificed 0.5 h after contact with the stranger to collect brains for counterstaining with antibodies (Figure 3B). Only the antagonist that reduced contacts with a stranger also increased the number of Fos-activated SST cells in dLS (Mann-Whitney NaCl vs. Manning *p* = 0.007 and NaCl vs. atosiban *p* = 0.1, Figure 3C). By contrast, it is the blockade of OXTR during familiarization that later reduced contacts with a stranger, while blocking AVPRs had no effects (one-way ANOVA NaCl vs. antagonists *p* = 0.02, Figure 3D). When counterstained after timed dissection as aforementioned (Figure 3E), only the antagonist that reduced contacts with a stranger also increased the number of Fos-activated SST cells in dLS (Mann-Whitney NaCl vs. Manning *p* = 0.2 and NaCl vs. atosiban *p* = 0.004, Figure 3F). These results suggest that AVPR and OXTR signal in dLS according to the degree of peer affiliation (Figure 3H). Yet, it remains unclear how this could influence treatment response. To address this point, we co-infused 10 pg AVP as therapeutic treatment (or NaCl as control) along with 10 nM of Manning compound or atosiban (or NaCl as control) in *Magel2*KO mice, according to the degree of peer affiliation. Only AVPR blockade *at the time* of AVP infusion (at novelty *before* habituation) reduced subsequent contacts with a stranger, as compared to AVP-injected KO controls (ANOVA NaCl vs. Manning at novelty *p* = 0.003 or familiarization *p* = 0.2, Figure 3G, *panel 3*). Blocking OXTR *1 h after* AVP infusion (at habituation) also impeded a following interaction with a stranger, while AVPR blockade *1 h after* AVP infusion (at habituation) had no effect (ANOVA *p* = 0.02, Figure 3G, *panels* 5 and 4, respectively). We concluded that both receptor subtypes are required to treat the *Magel2*KO phenotype. In combination with *ex vivo* physiological recordings (Figure 2), we predict that AVPR neurons target both the OXTR-sensitive and insensitive SST cells to regulate behavior (Figure 3H).

### *Magel2*KO mice failed to engage AVP and OXT PVN neurons as per degree of peer affiliation

**Figure 4:**
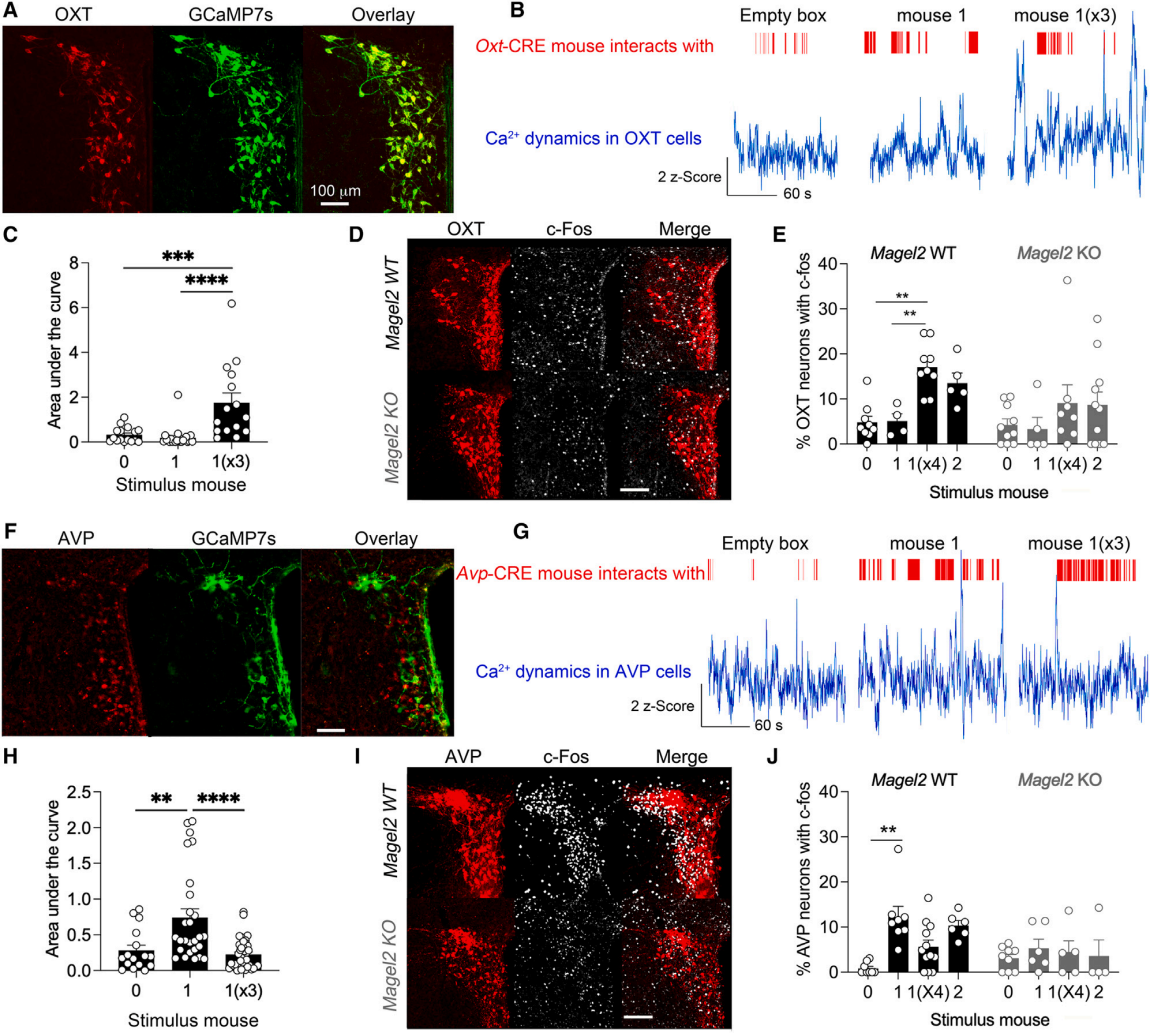
*Magel2*KO mice fail to engage AVP and OXT PVN cells as per degree of peer affiliation (A) Expression of CRE-dependent GCaMP7s in the PVN of *Oxt*-CRE mice. (B) Photometry traces during exposure to the empty box without/with the stimulus mouse once (×1) then repeatedly (×3). (C) Area under the curve during trial blocks. Means ± SEM, *n* events = 16 (empty), 22 (×1), 15 (×3) trials, and 3 WT mice. One-way ANOVA: *F*_(2,50)_ = 13.54 *p* < 0.0001, post hoc Tukey test and Cohen’s *d* effect size bigger during habituation (*d* = 1.4) than novelty (*d* = 0.3). See Figures S4A–S4D for controls. (D) Representative Fos induction in PVN after repeated exposure to the stimulus mouse. Scale, 100 μm. (E) Number of Fos-positive OXT cells in PVN with or without social stimulations. Brains were collected 0.5 h after the indicated stimulus trial. Means ± SEM, *n* WT mice = 9 (empty), 4 (×1), and 9 (×4), and *n* KO mice = 10 (empty), 5 (×1), and 8 (×4). Two-way ANOVA: effect of peer affiliation *F*_(2,41)_ = 9.26, *p* = 0.005, genotype *F*_(1,41)_ = 3.39, *p* = 0.07, post hoc Tukey test. Cohen’s *d* effect size bigger in PVN of WT than KO during novelty (*d* = 0.9) compared to SON of WT and KO during novelty (*d* = 0.3). See Figure S5A for analysis in the SON. (F) Expression of CRE-dependent GCaMP7s in the PVN of *Avp*-CRE mice. Scale, 100 μm. (G) Photometry traces during stimulation with the empty box without/with the stimulus mouse. (H) Area under the curve during social exploration. Means ± SEM, *n* = 16 empty, 27 (×1), 37 (×3) trials, and 3 WT mice. One-way ANOVA: *F*_(2,77)_ = 12.7, *p* < 0.0001, post hoc Tukey test and Cohen’s *d* effect size bigger during novelty (*d* = 1.16) than habituation (*d* = 0.2). See Figure S4E–S4H for controls. (I) Representative Fos induction in PVN after one exposure with the stimulus mouse. Scale, 100 μm. (J) Number of Fos-positive AVP cells in PVN without/with social stimulations. Brains were collected 0.5 h after the indicated stimulus trial. Means ± SEM, *n* WT mice = 8 (empty), 8 (once), and 12 (×4), and *n* KO mice = 9 (empty), 6 (once), and 5 (×4). Two-way ANOVA: effect of peer affiliation *F*_(2,42)_ = 8.2, *p* = 0.001, interaction with genotype *F*_(2,42)_ = 3.7, *p* = 0.031 post hoc Tukey test. Cohen’s *d* effect size bigger in PVN of WT than KO during novelty (*d* = 1.17) compared to SON of WT and KO during novelty (*d* = 0.06). See Figure S5B for analysis in the SON.

To examine how the degree of peer affiliation impacts the source of AVP and OXT delivered in dLS, we tracked the activity of neurons in the hypothalamus of either *Oxt*-CRE or *Avp*-CRE mice by photometry, as animals were presented with a stimulus mouse repeatedly to become familiar. A CRE-dependent virus was injected in the PVN to express GCaMP7s in OXT neurons and the optic fiber implanted atop (Figure 4A). Increased Ca^2+^ dynamics (Figure 4B) were observed upon third interaction with the same mouse (measured by the area under the curve, ANOVA repeat exposure vs. none *p* = 0.003, Figure 4C), which was not cued to turning away events (Figures S4A–S4D). However, it was more prominent within this 10-s period during social habituation compared to novelty or non-social exploration (Figures S4A–S4D). Brains were collected from *Magel2*KO mice 0.5 h after the last exposure as per degree of peer affiliation and counterstained with antibodies (Figure 4D). Strikingly, *Magel2*KO animals showed no increase in Fos-activated OXT cells in the PVN (Mann-Whitney WT vs. KO *p* = 0.01, Figure 4E), while the SON remained unaffected (Mann-Whitney WT vs. KO *p* = 0.5, Figure S5A). In contrast, AVP cells in the PVN expressing GCaMP7s (Figure 4F) showed changes of Ca^2+^ dynamics mostly during the 1^st^ exposure with a stranger (ANOVA one exposure vs. none *p* = 0.002, Figures 4G and 4H). Activity in AVP cells too was not cued to turning away events but was more prominent within this 10-s period during *social novelty* compared to habituation or non-social exploration (Figures S4E–S4H). Histological analyses (Figure 4I) unveiled that *Magel2* deficiency reduced the number of Fos-activated AVP cells in the PVN (Mann-Whitney WT vs. KO *p* = 0.04, Figure 4J) but not in the SON (Mann-Whitney WT vs. KO *p* = 0.3, Figure S5B). We concluded that *Magel2* deficiency interfered with the engagement of AVP and OXT neurons from the PVN as per degree of peer affiliation.

### Weak PVN→LS pathways in *Magel2*KO mice failed to correct social deficits with ChR2

**Figure 5:**
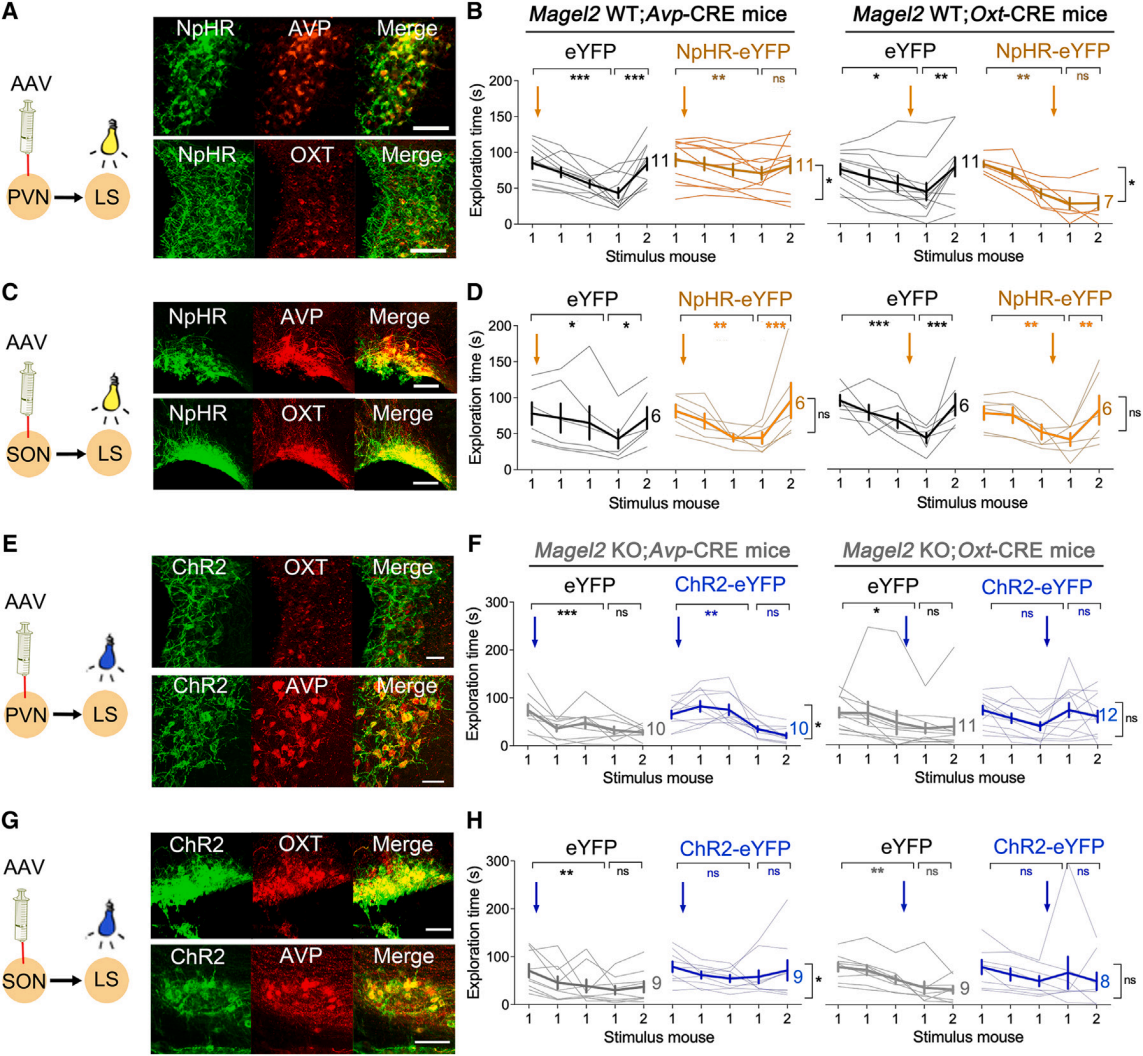
Weak PVN→LS pathways in *Magel2*KO mice fail to correct social deficits with ChR2 (A) Viral-mediated expression of DIO-NpHR3-eYFP in the PVN of *Magel2*WT*;Avp*-CRE (top) and *Magel2*WT*;Oxt*-CRE (bottom) mice. Scale, 40 μm. See Figure S5C for projecting axons in LS. (B) Optogenetic inhibition of the PVN→LS pathway during social novelty for AVP neurons or habituation for OXT neurons decreased the duration of contact with a stranger. Means ± SEM, *n* mice as indicated*.* Two-way ANOVA: trial × NpHR3 in *Magel2*WT*;Avp*-CRE mice (*F*_(4,80)_ = 3.2 *p* = 0.01) and *Magel2*WT*;Oxt*-CRE mice (*F*_(4,64)_ = 9.4 *p* < 0.0001), post hoc analysis with Dunnett test. NpHR blocks the effect size in *Avp*-CRE + YFP (*d* = 0.3 vs. *d* = 1.55). NpHR blocks the effect size in *Oxt*-CRE + YFP (*d* = 3.2 vs. *d* = 0.5). (C) Expression of DIO-NpHR3-eYFP in the SON of *Magel2*WT*;Avp*-CRE (top) and *Magel2*WT*;Oxt*-CRE (bottom) mice. Scale, 40 μm. (D) No effect of optogenetic silencing of the SON→LS pathways on the duration of social contacts. Means ± SEM, *n* mice as indicated*.* Two-way ANOVA: trial × NpHR3 in *Magel2*WT*;Avp*-CRE mice (*F*_(4,40)_ = 1.98 *p* = 0.1 and *d* = 0.1) and *Magel2*WT*;Oxt*-CRE mice (*F*_(4,40)_ = 0.4 *p* = 0.7 and *d* = 0.4). (E) Expression of DIO-ChR2-eYFP in the PVN of *Magel2*KO*;Avp*-CRE and *Magel2*KO*;Oxt*-CRE mice. Scale, 40 μm. (F) Optogenetic activation of the PVN→LS pathways increased social exploration but failed to correct social discrimination deficits in *Magel2*KO mice crossed with *Avp*-CRE or *Oxt*-CRE lines. Means ± SEM, *n* mice as indicated*.* Two-way ANOVA: trial × ChR2 in AVP neurons *F*_(4,72)_ = 6.4 *p* = 0.0002 and in OXT neurons *F*_(4,80)_ = 2.36 *p* = 0.05, post hoc analysis with Dunnett test. Cohen’s *d* effect size bigger with ChR2 (*d* = 0.77) than YFP (*d* = 0.2) in *Avp*-CRE mice but not in *Oxt*-CRE mice (ChR2 *d* = 0.2 vs. YFP *d* = 0.08). See Figures S5D and S5E for control optogenetic activation of the PVN→LS pathways during social novelty compared to habituation. (G) Expression of DIO-ChR2-eYFP in the SON of *Magel2*KO*;Avp*-CRE and *Magel2*KO*;Oxt*-CRE mice. Scale, 40 μm. (H) Effect of optogenetic activation of the SON→LS pathways in *Magel2*KO mice crossed with *Avp*-CRE or *Oxt*-CRE lines. Means ± SEM, *n* mice as indicated*.* Trial × ChR2 in *Magel2*KO*;Avp*-CRE mice (*F*_(4,64)_ = 0.68 *p* = 0.6 and *d* = 0.67) and *Magel2*KO*;Oxt*-CRE mice (*F*_(4,60)_ = 1.6 *p* = 0.18 and *d* = 0.0001).

To determine the behavioral relevance of the PVN→LS pathways as per degree of peer affiliation, we injected CRE-dependent virus in the PVN (or SON as control) of *Avp*-CRE and *Oxt*-CRE mice to express optogenes in, respectively, 71% and 62% of AVP and OXT cells that send projections in the LS (Figure S5C). We used the CRE-dependent halorhodopsin NpHR3-eYFP (or eYFP as control) to silence fibers in the LS with optic fibers implanted atop (Figures 5A and 5C). In WT mice, we found that stimulation of NpHR3 with continuous yellow light in the vasopressinergic PVN→LS pathway during novelty and the oxytocinergic PVN→LS pathway later during familiarization reduced contacts with a stranger (ANOVA YFP vs. NpHR in OXT cells *p* = 0.006 or AVP cells *p* = 0.02, Figure 5B). Remarkably, the silencing of either SON→LS pathways at either time point had no effect (ANOVA YFP vs. NpHR in OXT cells *p* = 0.9 or AVP cells *p* = 0.8, Figure 5D). Therefore, pathway-specific engagement of AVP and OXT in dLS is cued to the degree of peer affiliation.

Finally, to determine the strength of the PVN→LS pathways in *Magel2*KO mice, we made double transgenic animals with the *Avp*-CRE or *Oxt*-CRE lines for expressing CRE-dependent ChR2-eYFP (or eYFP as control) in the PVN (or SON as control) (Figures 5E and 5G). We previously characterized with opto-pharmacology in acute slices that OXT and AVP can be functionally released by hypothalamic-derived LS fibers stimulated by ChR2.^23^ *In vivo*, optogenetic activation of AVP fibers in the *Magel2*KO LS with pulsed blue light increased contacts with the 1^st^ stimulus mouse but not with the 2^nd^ (ANOVA YFP vs. ChR2 for mouse 1 *p* = 0.001 and mouse 2 *p* = 0.9, Figure 5F). In contrast, neither optogenetic activation of OXT fibers during habituation had any effect on stranger interaction (ANOVA YFP vs. ChR2 *p* = 0.8, Figure 5F), nor did activation of SON→LS pathways increase stranger contacts (ANOVA YFP vs. ChR2 in AVP cells *p* = 0.2 or OXT cells *p* = 0.4, Figure 5H). Finally, optogenetic activation of OXT fibers in the LS during social novelty, when it is not normally engaged specifically from the PVN (Figure S5D) or from all OXT pathways (Figure S5E), impaired social discrimination. Altogether, we concluded that confronting the loss of OXT in the *Magel2*KO model depends on AVP signaling, which is also cued to the degree of peer affiliation with another conspecific.

## Discussion

We characterized how the failure to disengage SST neurons from LS circuitry decreased sociability toward non-threatening strangers. *Magel2* deficiency impeded the modulation of SST cells by AVP and OXT normally delivered in the LS as per degree of peer affiliation. Blocking SST signaling was sufficient to correct the *Magel2*KO phenotype, perhaps by enabling exocytosis of OXT and AVP, which is blocked by SST via cAMP signaling in rodents.^37^^,^^38^ Another possible reason is that OXT and AVP signaling are co-dependent, and supplying one when the other is lacking may be ineffective. For instance, intra-septal infusion of AVP required OXT signaling to promote contacts with a stranger after social habituation, which was impaired in *Magel2*KO mice due to OXTR depletion in SST cells. Such dependency between neuropeptides evoked by peer affiliation may also explain why AVPR blockade during novelty affected contact with the stimulus mouse 2 but not with the mouse 1, albeit both are strangers to begin with. Deficits of social memory were also observed in double KO mice lacking AVPR1B and OXTR co-expressed in pyramidal neurons of CA2 hippocampal subfield upstream of the LS,^39^ further suggesting their co-dependent action.

Considering an SST approach to treatment, antagonism is safe and heavily used in clinical imaging,^40^ although chronic utilization might be poorly tolerated by the metabolism and affect appetite control.^41^ Future strategies will focus on how to target specific SST receptor subtypes for treating social deficits in neuropsychiatric diseases. Beyond SST, the present results explain why therapeutic strategies relying on the sole use of OXT or AVP might fail in the clinic. Given that a functional interplay between AVP and OXT signaling enables meeting with mates and strangers, the results strongly advise for the utilization of combinations of both to suppress the hyper-SST drive in *Magel2*-related diseases. Additionally, the methods used to assess the efficacy of treatment shall consider that neuropeptide effects are cued to experiential context. Here, we showed that the PVN→LS pathways supported sociability toward strangers in non-aversive context. In contrast, the SON→LS pathways support the acquisition of safety in socially aversive context.^23^ Both contexts should thus be used to assess primary outcome measures of treatments. For instance, functional imaging of septal nuclei in healthy human adults revealed activity in the LS specifically evoked by socially non-aversive tasks, and its modulation by both intranasal AVP and OXT,^42^ suggesting a limitation in context-dependent treatment efficacy.

SST neurons in the LS receive inputs locally and from distant regions in the hippocampus, locus coeruleus, periaqueductal gray, and hypothalamus for responding to diverse stressors.^29^^,^^43^ For instance, the hippocampus→LS pathway relayed by SST neurons is a key mediator of the approach-avoidance conflict.^44^^,^^45^ Additionally, the CA2→LS pathway inhibits aggression by pyramidal neurons equipped with AVPR1B but not AVPR1A,^46^ which is expressed in the post-synaptic LS GABAergic neurons.^23^ Mechanistically, we propose that AVP signals indirectly through GABA-A post-synaptic receptors that hyperpolarize SST cells while OXT signals through GABA-B post-synaptic receptors in SST cells as previously described in lamina II neurons of the spinal cord.^47^ Previously, post-synaptic GABA-B receptors were pharmacologically characterized in slices of dLS with the same dose of CGP35348 that we used and also with CGP55845 (100-fold more potent), while pre-synaptic GABA-B receptors were only sensitive to CGP55845.^48^ In contrast, AVPR1B from CA2 terminals is expected to facilitate excitatory transmission in dLS,^46^ which could explain the cryptic excitatory synaptic current activity in SST cells evoked by AVP in the presence of TTX. Effectors of OXTR signaling downstream of post-synaptic GABA-B could possibly involve: (1) TRPV1 cationic channel inhibition that decreased lamina II neurons activity in the spinal cord^47^; (2) Cav1.2 L-type channel inhibition that blocked LTP at excitatory synapses in SST hippocampal cells^49^ or that suppressed OXT and AVP neuron firing activity in the hypothalamus and possibly in long-range axons^50^; and (3) KCC2 Cl^−^/K^+^ cotransporter that sets [Cl^−^]_i_ in mature neurons and thereby establishes the driving force for the Cl^−^-permeable GABA-A receptors.^51^ All three pathways could be altered by *Magel2* deficiency. For instance, *Magel2* deficiency reduced KCC2 levels in the adult hippocampus, causing lesser Cl^−^ extrusion, more [Cl^−^]_i_ that changed the reversal potential for GABA-A receptors, and thus its inhibitory effect.^52^ Besides, KCC2 expression is facilitated by OXT signaling in the developing brain.^53^ Apart from afferent inputs, efferent projections of SST neurons locally within LS and long-range to the hypothalamus and midbrain control a brain-and-body endocrine response to offensive/defensive behaviors,^29^ and reward via dopaminergic outputs.^53^ AVP/OXT administration on such output networks could also be therapeutic—for instance, counteracting the loss of OXT and AVP support in the LS prevents aggression in *Magel2*KO, similar to tantrums in *Magel2*-related diseases, possibly through the LS→lateral hypothalamus pathway.^23^

Based on current knowledge, a hyper-SST drive in *Magel2*-related diseases might correspond with impaired endocrine response to stress. It has been suggested that patients with Prader-Willi syndrome have central adrenal insufficiency under stressful conditions,^54^ delayed peak response of cortisol to insulin challenge,^55^ and small-sized adrenal glands in autopsies.^56^ Consistently, there are differentially methylated regions in genes involved in cortisol synthesis, Cushing syndrome, and endocrine resistance in blood cells of patients with Prader-Willi syndrome compared to neurotypical controls.^57^ Mice lacking *Magel2* display excessive basal plasma corticosterone levels but normal stress-reactive levels.^58^ Given that AVP facilitates CRH release, which controls corticosterone secretion under stress,^59^ it is possible that a weakening of AVP release in *Magel2*KO mice prevents this response. It is also plausible that a weakening of the OXT endocrine response in *Magel2*-related disease, which blocks ACTH release that further controls corticosterone secretion, could prevent the buffering effect of social interactions on the stress response.^60^ Thus, future studies targeting neuropeptide-based interventions in *Magel2-*related diseases should also integrate the stress-response axis when designing the treatment framework to improve social behaviors.

### Limitations of the study

Although *Magel2*KO mice are best known as model of Prader-Willi and Schaaf-Yang syndromes, present results should be validated in other disease models featuring social deficits related to ASD as well as in patients. Neuropeptide release has not been monitored in this study but previous microdialysis in the LS showed that AVP and OXT releases are cued to distinct peer affiliations.^32^^,^^33^ Furthermore, our use of a non-selective SST antagonist is due to the fact that 4 SSTR subtypes are expressed in the LS,^31^ demanding future studies to investigate the effect of each subtype. Additionally, atosiban is a partial OXTR antagonist at Gq but not Gi-mediated signaling,^34^^,^^36^ suggesting that OXT behavioral effects depend on the Gq pathway in the LS as in other brain regions.^35^ Future experiments will use an unbiased antagonist or a biased Gq-dependent agonist to distinguish between signaling cascades. One major effect of *Magel2* deficiency is OXTR depletion in SST cells. We have not re-expressed OXTR in the LS of *Magel2*KO mice because such an experiment has already been done in depleted brain cells of neurotypical mice which modified behaviors.^61^^,^^62^ Besides, neonatal injections of OXT alleviated the *Magel2*KO phenotype in adults with concurrent reduction of SST levels and elevation of OXTR expression in the brain.^52^ Additionally, we have not studied females, but previous genetic deletion of OXTR in SST neurons of the prefrontal cortex impaired social preference in neurotypical female mice,^63^ which is consistent with the OXTR depletion effect of *Magel2* inactivation in males. Neither have we looked for the mechanism underlying OXTR depletion, although a previous study indicates that variations in OXTR expression in the brain can be attributed to a polymorphism in its promoter, and OXTR mosaic expression shaped by epigenetic and environmental factors, predicting behavioral variations.^64^ Future studies will investigate the interaction of genotype with epigenetic and environmental factors. Despite these limitations, we conclude that SST acted as an accelerator, and OXT and AVP as brakes on LS neurons suppressing sociability. While targeting the brakes has been mainly disappointing and conflicting, moderating the accelerator represents a viable alternative for treating social deficits in neuropsychiatric diseases.

## Resource availability

### Lead contact

Further information and requests for resources and reagents should be directed to and will be fulfilled by the lead contact, Freddy Jeanneteau (freddy.jeanneteau@igf.cnrs.fr).

### Materials availability

Tools generated in this study are available from the lead contact upon request with a materials transfer agreement.

### Data and code availability

• Accession number of data https://doi.org/10.5281/zenodo.10678960 without restriction.
• This paper does not report original code.
• Any additional information required to reanalyze the data reported in this paper is available from the lead contact upon request.

## Acknowledgments

We thank H. Gainer (NIH, USA) for antibodies and A. Besnard (IGF, France) for critical comments.

This study was funded by Foundation pour la Recherche Médicale Equipe-FRM 2018 (F.J.).

## Author contributions

Conceptualization and methodology, A.M.B., P. Chakraborty., and F.J.; investigation and formal analyses, A.M.B., Y.D., P. Chakraborty., F.J., G.G., and E.M.A.; resource, F.M., A.F., and P.F.; writing, F.J.; editing, P. Chakraborty.; administration, supervision, and funding acquisition, F.J., M.G.D., and P. Colson.

## Declaration of interests

The authors declare no competing interests.

## STAR★Methods

### Key resources table

**Table:** 

REAGENT or RESOURCE	SOURCE	IDENTIFIER
**Antibodies**		

Somatostatin (SST)	Abcam	Cat# ab30788, RRID:AB_778010
SST	SantaCruz Biotechnologies H-11	Cat# sc-74556, RRID:AB_2271061
GFP	Abcam	Cat# ab13970, RRID:AB_300798
c-Fos (9F6)	Cell Signaling Technology	Cat# 2250, RRID:AB_2247211
c-Fos (E8)	Santa Cruz Laboratories	Cat# sc-166940, RRID:AB_10609634
NPI-OXT	H. Gainer at NIH, USA	Cat# PS-38, RRID:AB_2315026
NPII-AVP	H. Gainer at NIH, USA	Cat# PS41, RRID:AB_2313960
Fab anti mouse IgG	Abliance	Cat# BI 1013C
Goat anti-rabbit Alexa Fluor 488/594/647	Thermo Fisher Scientific	Cat#A-11034/11037/21244; RRID: AB_2576217, RRID: AB_2534095, RRID: AB_2535812
Goat anti-mouse Alexa Fluor 488/594/647	Thermo Fisher Scientific	Cat#A-11029/11032/21236; RRID: AB_2534088; RRID: AB_2534091; RRID: AB_141725

**Chemicals, peptides, and recombinant proteins**		

Arg-vasopressin	Merck	CAS#113-79-1
TGOT	Merck	CAS# 60786-59-6
Atosiban	Merck	CAS#90779-69-4
Manning Compound (MC)	Bachem	CAS#73168-24-8
SR95531 (GABAzine)	Merck	CAS# 104104-50-9
Tetrodotoxin (TTX)	Merck	CAS# 4368-28-9
6-Cyano-7nitroquinoxaline-8 2,3-dione (CNQX)	Merck	CAS# 115066-14-3
CGP35348	Merck	CAS# 160415-07-6
SST14	Merck	CAS# 38916-34-6
cyclosomatostatin	Merck	CAS# 38916-34-6
Alexa 594-cadaverine	Life Technology	Cat# A30678
d[L(Alexa Fluor 647)8]VP	Dromard et al. Biological Psychiatry 2024	N/A
Desamino-Cys,^1^ Lys^8^]Vasopressin	Bachem	CAS#16679-58-6
Paraformaldehyde	Merck	Cat#P6148
Alexa 647 carboxylic acid succinimidyl ester	ThermoFisher Scientific	CAT#A37573
pentobarbital	Ceva Santé Animale	QN51AA01
xylazine	Ceva Santé Animale	QN05CM92
Ketamine	Ceva Santé Animale	QN01AX03

**Deposited data**		

Supporting data	Zenodo	https://doi.org/10.5281/zenodo.10666765

**Experimental models: Organisms/strains**		

AAV1 EF1a:DIO-eNpHR3.0-eYFP; WPRE:hGH	Univ Pennsylvania, USA	N/A
AAV1 EF1a:DIO-ChR2-eYFP; WPRE:hGH	Univ Pennsylvania, USA	N/A
AAV1 EF1a:DIO-eYFP; WPRE:hGH	Univ Pennsylvania, USA	N/A
AAV2/5 pGC-CAG-FLEX-jGCaMP7s-WPRE:hGH	CERVO, Canada	N/A
Cg-Sst^tm2.1^(cre)^Zjh/Mwar^J	Jackson laboratories	Cat#028864; RRID: IMSR_JAX:028864
Oxt^tm1.1^(CRE)^Dolsn^/J	Jackson laboratories	Cat# 024234; RRID: IMSR_JAX:024234
Avp^tm1.1^(cre)^Hze^/J	Jackson laboratories	Cat#023530; RRID: IMSR_JAX:023530
Magel2^tm1.1Mus^/J	F. Muscatelli, INMED, France	MGI:4849506
**B6.Cg-Gt(ROSA)26Sor**^**tm27.1(CAG-COP4∗H134R/tdTomato)Hze**^**/J**	Jackson laboratories	RRID:IMSR_JAX:012567
C57BL6J	Charles River	Cat#000664; RRID: IMSR_JAX:000664

**Oligonucleotides**		

cre Tg allele forward 5′-TCTGTCCGTTTGCCGGTCGT-3′	Dromard et al. Biological Psychiatry 2024	N/A
cre Tg allele reverse 5′-AGACCGCGCGCCTGAAGATA-3′	Dromard et al. Biological Psychiatry 2024	N/A
AVP-cre WT allele forward 5′-GAGTCCGTGGATTCTGCCAA-3′	Borie et al. JCI 2021	N/A
AVP-cre WT allele reverse 5′-CTATGCACGACTTCGGGTGT-3′	Borie et al. JCI 2021	N/A
OXT-cre WT allele forward 5′-CTCAGAACACTGACCCATTTCTCTT-3′	Dromard et al. Biological Psychiatry 2024	N/A
OXT-cre WT allele reverse 5′-CCGACAATTAGACACCAGTCAAG-3′	Dromard et al. Biological Psychiatry 2024	N/A
SST-cre Tg Mutant Forward 5′-TCAGGTACATGGATCCACTAGTTCT-3′	Jackson laboratories	Primer 53428
SST-cre Tg allele Common 5′-AGTCAAACGCTTGCTCTTCA-3′	Jackson laboratories	Primer 53430
SST-cre WT allele Wild type Forward 5′-GAGGTCTGCCAACTCGAAC-3′	Jackson laboratories	Primer 53429
Magel2 KO allele forward 5′-TGCTTCCTGCCCTTCAGTTAC-3′	Meziane et al. ^12^ Biol Psy 2015	N/A
Magel2 KO allele reverse 5′-GCTTATCGATACCGTCGACCTC-3′	Meziane et al. ^12^ Biol Psy 2015	N/A
Magel2 WT allele forward 5′-GTCACACACCCATTCGACCT-3′	Meziane et al. ^12^ Biol Psy 2015	N/A
Magel2 WT allele reverse 5′-TACCCTCGGGAGCAGTAGAC-3′	Meziane et al. ^12^ Biol Psy 2015	N/A
Ai27-D WT allele 5′-AAGGGAGCTGCAGTGGAGTA-3′	Jackson laboratories	oIMR9020
Ai27-D WT allele 5′-CCGAAAATC TGTGGGAAGTC-3′	Jackson laboratories	oIMR9021
Ai27-D WPRE 5′-GGCATTAAAGCAGCGTATCC-3′	Jackson laboratories	oIMR9103
tdTomato allele 5′-CTGTTCCTGTACGGC-3′	Jackson laboratories	oIMR9105

**Software and algorithms**		

Graphpad prism 9.0	http://graphpad.com	SCR_002798
Adobe Creative Suite 6 (Photoshop, Illustrator)	https://www.adobe.com/de/products/cs6.html	RRID:SCR_010279
Fiji ImageJ	http://imagej.net/Fiji	SCR_003070
Behavioral Observation Research Interactive Software (BORIS)v. 7.9.4	https://www.boris.unito.it/	RRID:SCR_025700
DORIC Neuroscience studio	http://doriclenses.com/life-sciences/software/955-doric-neuroscience-studio.html	RRID:SCR_018569
MATLAB script	https://github.com/PHYSIOPATHOLOGIE-RYTHME-CARDIAQUE/Photometry)	This paper
PMATv1-3	https://github.com/djamesbarker/pMAT	RRID:SCR_022570
pClamp software	http://www.moleculardevices.com/products/software/pclamp.html	RRID:SCR_011323

**Other**		

Fluoromount	ThermoFisher Scientific	CAT# 00-4958-02
Dental cement	Paladur, Henry Schein	CAT#203097
Mandrin double pas de projection	Phymed	CAT#C235DCS-5/3/0
Small dust cap	Phymed	CAT# 303DC/1
Canule interne double projection 1mm	–	CAT# C235IS-5/3/1
Guide canule double 26G	Phymed	CAT# C235GS-5-0,8/3 0.8mm 3mm
Branching Fiberoptic Patchcord	Doric lenses	CAT# BFP(2)_200/220/900-0.53_1m_FCM-GS0
Dual fiber optic cannula with Guiding Socket	Doric lenses	CAT# DFC_200/245-0.53_3.5mm_GS0.8_FLT
Luminometer	Thorlab	N/A
LED	Doric Lenses	LEDFRJ_B/A_FC
LED driver console	Doric Lenses	LEDRVP_2CH_1000
Monofiber optic cannula	Doric Lenses	MFC_400/430-0.66_MF1.25_FLT

### Experimental model and study participant details

#### Study approval

All procedures were performed using protocols reviewed and approved by the Ethics Committee of the University of Montpellier and the French ministry of research and agriculture (Approved protocol numbers: CEEA-APAFIS-1576-18932, CEEA-APAFIS-1770-28535, CEEA-APAFIS-1621-21218).

#### Animal studies

Mice were kept in-group of 2–4 under standard pathogen-free laboratory conditions in transparent polycarbonate cages (22 × 30 × 16 cm) (12/12 light/dark cycle, 22°C, 60% humidity, standard food and water *ad libitum*). Age/weight-matched mice were used throughout the study. All procedures were performed between 8:00 and 15:00-h according to the ARRIVE guidelines. Genetic lines were all backcrossed more than 10 times with C57Bl6J from Janvier labs and are as follow: *Oxt*-Cre (Oxt^tm1.1^(Cre)^Dolsn^/J), *Avp*-Cre (Avp^tm1.1^(Cre)^Hze^/J), *Sst*-Cre (Cg-Sst^tm2.1^(Cre)^Zjh/Mwar^J)), (Ai27)RCLhChR2(H134R)/tdT-D from Jackson labs and *Magel2* KO mice (*Magel2*^tm1.1Mus^/J). *Magel2* gene is paternally (p) expressed and maternally (m) imprinted such that heterozygotes can be knockouts when the null allele is paternal (-p). All experiments were performed with heterozygote *Magel2*+m/-p mice as KO and *Magel2*+m/+p mice as WT. Littermates were randomly allocated to experimental groups prior to experiments. Experimenters were blinded to genotypes and treatment conditions for data collection and analysis. All lines are openly available from commercial vendors except for the *Magel2* KO mice subject to MTA with F. Muscatelli.

### Method details

#### Behavioral test

Mice (4–6 months) were habituated to manipulation by the same experimenter a week before. A stimulus box where the juvenile would be introduced, was placed in the arena (24 cm diameter) accommodated with clean litter before the beginning of each experiment. Non-sibling male juveniles (21–31 days) were used as stimulus mice as previously.^65^ We have used juvenile males to avoid agonistic behaviors frequent between adult males but absent between an adult male and a juvenile male. The juveniles were randomly specified as stimulus mouse 1 or 2. The task consists of a 5-min interaction repeated 4 times (1(x4) with the first juvenile spaced by 20-min intervals to become familiar followed by 5-min interaction with another juvenile during trial 5. There are several variants of this task with shorter inter-trial intervals (5 min).^66^ We chose inter-trial duration that lasted 4 times longer than trials to make clear separation between social exposures. Typically, mice reduced exploration of the first juvenile as it became familiar and regain full interest with the second juvenile stranger. We used this test because *Magel2* KO mice failed to regain interest with the second juvenile stranger. This particular behavioral sequence allowed us to test *in vivo* physiological effects of OXT and AVP secretions as per degree of peer affiliation. An experimenter blind to the groups counted the duration of contacts with the juveniles during each trial. Animals were included in analyses based on *a priori* criterion of performance during trial 1 (Value within the range +/− 2 SD from the mean). For instance, animals were not excluded if performance exceeded these thresholds in the subsequent trials. However, we excluded mice that explored the first juvenile on trial 1 less than 15 s. Behavioral results were replicated by several experimenters to test independently the effects of pharmacological treatments and opsins. Sample size in this test was based on previous experiments done with the same design in *Magel2* KO mice.^11^ We used the Shapiro-Wilk and Kolmogorov-Smirnov tests to verify that data met the assumption of normal distribution. We used two-way ANOVA to compare data across trials with dependent variables (genotype, NpHR, ChR2, antagonist, agonist).

#### Stereotaxic surgery

Surgeries were performed under anesthesia with ketamine (6.6 g/kg) and xylazine (1.3 g/kg). Virus injections were administered to mice aged 3–4 months old with their heads were fixed in a stereotaxic injection frame under aseptic conditions and the animals were maintained their body temperature with a heating pad. All skull measurements were made relative to bregma. Animals were given anti-inflammatory medication (meloxicam, 10 mg/kg) and monitored daily for 3 weeks. For all virus injection, we used a Hamilton syringe loaded with 500 nL (2x10^11^/mL) AAV with mixed serotypes 1/2 (UPENN, USA and CERVO, CA, key resource table) and coupled with a microinjector at a rate of 50 nL/min to reach the PVN (AP -0.9 mm, ML +/−0.2 mm, DV -4.5 mm), SON (AP -0.56 mm, ML +/−1.13 mm, DV -5.45 mm) or dLS (AP 0.3 mm, ML +/−0.3 mm, DV -2.5 mm). Following the viral injection, the syringe was held at the site for 10 min to allow diffusion of the virus. Then the incision was sutured and mice recovered in a clean cage on a heating pad. All viral vectors were aliquoted and stored at −80°C until used. Recombination of transgenes was verified in postmortem brain sections and antibodies for the target cells. Infection rates were as follows. NpHR3.0: AVP^PVN^ (71%), AVP^SON^ (78%); OXT^PVN^ (62%), OXT^SON^ (81%); SST^LS^ (88%); ChR2: AVP^Ai27−D^ (88%), OXT^Ai27−D^ (93%), AVP^PVN^ (68%), OXT^PVN^ (64%), SST^LS^ (71%); GCaMP7s: SST^LS^ (77%). All viruses are openly available from vendors.

#### Optogenetic studies

Intensity of LED stimulation for each optic fiber (Doric lenses, 0.53 NA) was calibrated to deliver about 2 mW at the tip with a luminometer (Thorlab). Optic fibers were implanted bilaterally in dLS using aforementioned coordinates. At least 3 weeks post-surgery, stimulations with a LED system controlled by a digital driver console (DORIC lenses, CA) were as follow: continuous stimulation at 561 nm (NpHR3.0), and 20 Hz, 5 ms at 473 nm (ChR2) in AVP neurons or 30 Hz for 10 ms in OXT neurons or 15 Hz for 20 ms in SST neurons as previously.^23^ Opsin-expressing groups and eYFP-injected controls were exposed to the same stimulation protocols by an experimenter blind to the groups. We previously used Manning Compound and Atosiban to verify the specificity of opsin-evoked effects on OXT and AVP signaling in the LS.^23^ Implantation sites were verified in postmortem sections. We have used two-way ANOVA analyses to compare between the effect of opsins and eYFP controls across behavioral trials.

#### Photometry

Surgeries were performed under anesthesia with ketamine (6.6 g/kg) and xylazine (1.3 g/kg) to inject 500 nL of virus (AAV2/5-Syn-jGCaMP7s, CERVO) to mice aged 3–4 months old as previously described. The virus was injected unilaterally and an optic monofiber implanted in the LS (AP + 0.3 mm, ML +/− 0.3 mm, DV -2.5 mm) of *Magel2*KO*;Sst*-Cre mice or in the PVN (AP -0.9 mm, ML +/−0.2 mm, DV -4.5 mm) of *Avp*-CRE mice or *Oxt*-CRE mice. Three weeks after surgery, freely moving animals were connected to a low-autofluorescence fiber optic patchcord connected to a rotary joint and cLEDs emitting sinusoidal signals (GCaMP, 465 nm, and isosbestic, 405 nm) were connected to a fluorescence minicube for photostimulation (DORIC lenses, Quebec, CA). Signals were acquired simultaneously with behavioral data using DORIC Neuroscience studio, processed with a low-pass filter (12 Hz), and demodulated in real time with a lock-in mode of acquisition. GCaMP and isosbestic signals were smoothed using loess and linearized using a moving minima algorithm. GCaMP signal was fit to the isosbestic using linear regression. ΔF/F was calculated by subtracting the fitted isosbestic from the fitted GCaMP, followed by dividing by the fitted isosbestic. *Z* score was computed and fluorescence changes in the positive area under spikes (>+3 SD from baseline) and the width at the base of spikes were analyzed with PMATv1-3 and custom-written MATLAB scripts as previously described.^21^ The script is openly available on Jithub or on demand. We have used one-way ANOVA analyses to determine the changes of fluorescence between timepoints in either OXT neurons or AVP neurons. We used the Shapiro-Wilk and Kolmogorov-Smirnov tests to verify that data met the assumption of normal distribution. We have used two-way ANOVA analyses to determine changes of fluorescence as a function of dependent variables (genotype and degree of peer affiliation). This was done for comparing changes of the area under the curves and the width at the base of spikes.

#### Intracerebral infusions

Bilateral cannula guides (Phymep) were implanted in LS at the aforementioned coordinates using stereotaxic surgical methods previously described. Drugs were injected with injectors connected to two 1 μL Hamilton syringes controlled by a pump (WPI). Injections of 900 nL/hemisphere (100 nL/min) started 5 min before the indicated trial and lasted 9 min so it lasted throughout the indicated trial. Mice were injected with 0.9% NaCl, 10 nM Atosiban (Merck), 10 nM Manning compound (Bachem), 30 μM AVP, 1 nM SST14 and 1 μM cyclosomatostatin (Sigma Aldrich) based on published selectivity profiles for rodent receptors.^67^ Injection of 50 μM Alexa 594-cadaverine (Life Technology) visualized the diffusion area. Given that AVP concentration increases in LS during novelty and OXT concentration increases in LS during familiarization, injections were performed as per degree of peer affiliation. We chose the first trial for novelty and the third trial for familiarization. For co-injections during novelty, the agonist and antagonist were mixed together. For subsequent injections, the agonist was injected during novelty and the syringe reloaded with the antagonist for the injection during habituation. All injections were compared with NaCl-injected controls for either the co-injection or the subsequent injection protocols. We used the Shapiro-Wilk and Kolmogorov-Smirnov tests to verify that data met the assumption of normal distribution. We used two-way ANOVA to compared the effects of a pharmacological treatment against NaCl across behavioral trials.

#### Patch-clamp

Animals (2–4 months old males) were anesthetized using isoflurane and quickly decapitated. Coronal slices were cut with a microtome (Campden Instr.) at 300 μm in ice-cold solution (in mM: 10 NaCl, 1.2 KCl, 26 NaHCO3, 15 glucose, 1.2 KH2PO4, 1 CaCl2, 2 MgCl2, 195 sucrose; osmolality adjusted to 300 mOsmol/L; pH = 7.4, 95% O2 and 5% CO2) and allowed to recover for 1h at 37°C in the recording chamber perfused at 1 mL/min with artificial cerebrospinal fluid (ACSF). Ag/AgCl electrodes inserted in a borosillicated glass (4–6 Ohm resistance) containing intracellular medium (in mM: 9 KCl, 130 KMeSO3, 8 NaCl, 1 MgCl2, 0.1 EGTA/Na, 10 HEPES/NaOH, 2 pyruvate, 2 malate, 0.5 NaH2PO4, 0.5 cAMP, 2 ATP-Mg, 0.5 GTP-Tris, 14 phosphocreatine, 0.1 leupeptine) were connected to the pre-amplifier. LS neurons were visualized using Normarsky contrast microscopy and randomly recorded from dLS. YFP-positive axon fibers from hypothalamus into the LS and cadaverine-filled cells were identified with a microscope equipped with GFP or RFP filter (Olympus, BX-51WI). Voltage-clamp recordings were acquired in whole-cell configuration at 10 kHz through an axopatch-200B amplifier (Axon instruments) connected to a digitizer and processed with Clampfitv9 (Molecular Devices). We fixed the [Cl^−^]_i_ to set the inversion potential E_Cl_ at −80 mV and recorded at −45 mV to distinguish between excitatory synaptic currents (EPSC) and inhibitory synaptic currents (IPSC). Alexa-Fluor-488-cadaverine (Life technology, 50 μM) was added to the intracellular medium of the pipette and counterstained with SST antibodies. All cells inhibited by TGOT (0.1 μM)/AVP (1 μM) bath-applied (2 min) are GABAergic SST neurons. CGP35348 (100 μM) and GABAzine (1 μM) were bath-applied during the entire period with TGOT/AVP to discriminate between GABA-A and GABA-B mediated currents. The sodium channel blocker TTX (0.3 μM) was bath-applied during the entire period with TGOT/AVP to test for spontaneous network activity on synaptic transmission. Frequency of action potentials was measured for each cell across the entire recording, expressed a % baseline for comparison between cell types. We used Wilcoxon test to determine significant change from baseline post-stimulation. Cells with less than 10% change from baseline on consecutive timepoints were considered insensitive while the others persistently deviating from baseline were pooled with the responding groups. We used two-way ANOVA to compared the responses of TGOT and AVP in the responding and insensitive cells. We specifically chose 20 min inter-stimulation intervals with AVP and TGOT to relate with the behavioral sequence used to study peer affiliation. The order of AVP and TGOT applications were randomized for each cell.

#### d[Lys(Alexa 647)^8^]VP binding

The affinity of d[Lys(Alexa 647)^8^]VP was previously determined by competitive binding assays with 1 nM [^3^H]AVP (PerkinElmer) on membrane preparations with recombinant receptors *in vitro*.^23^ Its affinity is 54, 205 and 2796 times higher for OXTR than for AVPR1B, AVPR1A and AVPR2, respectively. Binding of d[Lys(Alexa 647)^8^]VP is not specific in fixed tissue preparation. That’s why, binding must be done on live tissue either via *in vivo* infusion as previously^23^ or *ex vivo* application like in this study. To this end, acute slices were maintained at 12°C in ACSF and perfused at 1 mL/min with ACSF containing 150 nM d[Lys(Alexa Fluor 647)8]VP with Manning compound (5 μM) (non-specific) and without (total binding) for 1h, followed by 3 rinses and fixation with 4% PFA. Sections were counterstained with SST antibodies (Abcam), imaged with an epifluorescent microscope (ImagerZ1, Zeiss) and the number of double labeled cells counted in dLS using ImageJ. This experiment was replicated twice and data averaged. We used non-parametric analysis with a 2-sided unpaired t-test to compare data from WT and KO sections.

#### Fos-mapping

Deeply anesthetized mice (pentobarbital 50 mg/kg) were perfused with transcardiac ice-cold 0.9% NaCl and 4% PFA. The brains were removed and postfixed by immersion in the same fixative for 24 h at 4°C. Brains from *Sst*-CRE mice were collected 1 h after exposure with a stranger. In other experiments, brains were collected 0.5 h after the last peer affiliation trial to determine the effect of distinct peer affiliations. We used a vibratome for sectioning (50 μm thickness). Sections were blocked for 2 h in PBS-1X containing 5% bovine serum albumin, 3% normal donkey serum and 0.1% Triton X-100. Then the sections were incubated with the primary rabbit polyclonal antibodies against c-Fos (Cell Signaling) and for double staining with NPI-OXT, NPII-AVP mouse monoclonal antibodies (H. Gainer, NIH) or SST rat polyclonal antibodies (Abcam) or SST mouse monoclonal antibodies (SantaCruz Biotechnologies) for 48 h at 4°C. Sections were rinsed 5 times with PBS-1X, 0.1% Triton X-100 at least 10 min each. Alexa-Fluor-conjugated secondary antibodies (1:2,000 ThermoFisher Scientific) were incubated for 2 h at 25°C in PBS-1X, 0.1% Triton X-100. Sections were rinsed 5 times with PBS-1X, 0.1% Triton X-100 at least 10 min each. Sections were mounted with fluoromount and stored at 4°C. Images were acquired with an epifluorescence microscope (ImagerZ1, Carl Zeiss). Cells were counted in dLS by an experimenter blind to the groups. Data were normalized to the surface of the region and averaged between groups. We used a non-parametric analysis with a 2-sided unpaired Mann-Whitney test to compare one variable in 2 groups (genotype, responding vs. insensitive cells). Data that met the assumption of normal distribution with the Shapiro-Wilk and Kolmogorov-Smirnov test were analyzed with one-way ANOVA to compare the effect of pharmacological interventions on the duration of social contacts or with two-way ANOVA to compare dependent variables (genotype with peer affiliation).

### Quantifications and statistical analysis

Prism 9 (GraphPad, CA) and MATLAB (Mathworks) were used for statistical analysis. No statistical methods were used to pre-determine sample sizes. Sample size was chosen to ensure 80% power with α = 0.05 based on previous experiments with the same tests. Data were presented as means ± SEM, unless otherwise stated in the figure legends. The normality test was performed by the Shapiro-Wilk test. Non-parametric two-sided Mann-Whitney test was used for two-sided unpaired comparisons of the means and Wilcoxon test for two-sided variance comparisons. ANOVA was used for multiple factorial comparisons of the means followed by Tukey, Dunnett or Sidak’s multiple comparison test as indicated. Proportions were compared with Chi2 test. Details of particular statistical analysis can be found in see Table S1. The use of asterisks indicates statistical significance (∗*p* < 0.05, ∗∗*p* < 0.01, ∗∗∗*p* < 0.001, ∗∗∗∗*p* < 0.0001).

## Supplemental information

Document S1. Figures S1–S5

Table S1. Details of statistical analyses The table includes sample size, effect size and tests utilized, related to STAR Methods

Document S2. Article plus supplemental information
