# Supplementary material for: Neuropeptide therapeutics to repress lateral septum neurons that disable sociability in an autism mouse model
Source: Cell Rep Med. 2024 Oct 17;5(11):101781. doi: 10.1016/j.xcrm.2024.101781 (PMC11604546; doi:10.1016/j.xcrm.2024.101781)
Supplement: Document S1. Figures S1–S5 [file mmc1.pdf]

Cell Reports Medicine, Volume *5*

Supplemental information

Neuropeptide therapeutics to repress lateral septum neurons that disable sociability in an autism mouse model

Amélie M. Borie, Yann Dromard, Prabahan Chakraborty, Pierre Fontanaud, Emilie M. Andre, Amaury François, Pascal Colson, Françoise Muscatelli, Gilles Guillon, Michel G. Desarménien, and Freddy Jeanneteau

Supplemental Figures

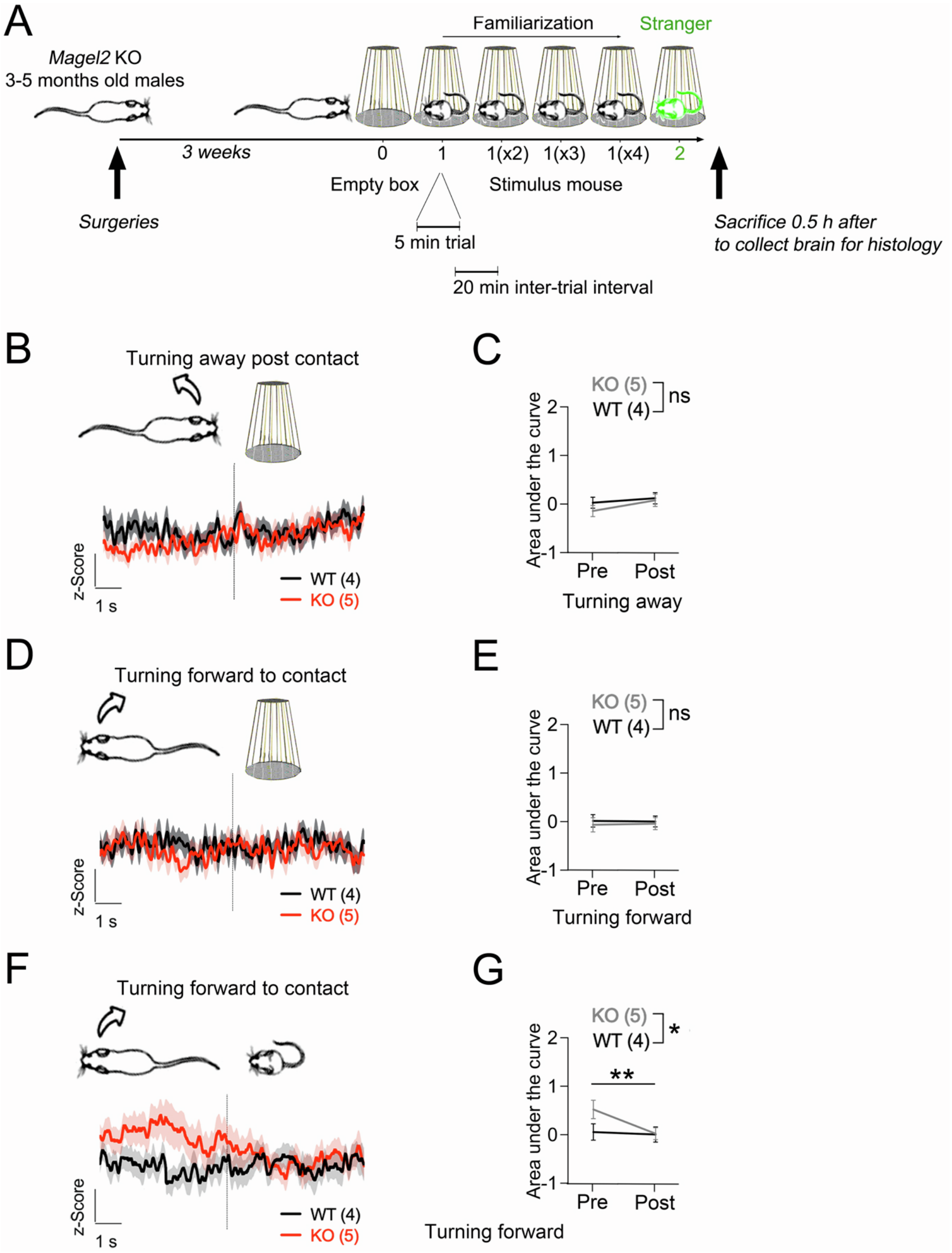

Figure S1. No change of Ca^{2+} dynamics in SST cells of dLS when exploring an object as when turning forward to contact with the stimulus mouse (related to Figure 1).

(A) Experimental timeline: Task consists of 6 consecutive trials to explore a stimulus box without or with a mate or a stranger. Stimulus mice are same-sex juveniles to avoid aggression and socio-sexual bias. Intervals are longer than trials to create a familiarization sequence on repetition of interactions with the same juvenile interrupted by the presentation of a stranger. *Magel2*KO mice have deficits of preference for novelty and forebrain activity in the theta band unveiled by this task [11]. Typically, mice reduced exploration of the first juvenile as it became familiar and regain full interest with the stranger, which *Magel2*KO mice failed to do. Mice were sacrificed 0.5 h after the last

trial for Fos-mapping. It reflects the sum of neuronal activity throughout the task that lasted 2.5 h (See Figure 1G). To capture the effect of pharmacological injections on Fos-induction, brains were also collected 0.5 h after the last trial (See Figure 3). To capture peak Fos-induction triggered by a specific trial, brains were collected 0.5 h after the indicated trial (see Figure 4E,J).
(B) Ca^{2+} dynamics when subjects turn away from the empty prison. Means ±SEM of N=41 events in 4 WT mice and 34 in 5 KO mice.
(C) Change of Ca^{2+} dynamics before and after turning away events. Means ±SEM of N=41 events in 4 WT mice and 34 in 5 KO mice. Two-way ANOVA: Effect of genotype $F_{(1,73)}=0.5$, $p=0.47$; effect of pre vs post $F_{(1,73)}=3$, $p=0.067$, effect of interaction $F_{(1,73)}=0.6$, $p=0.4$.
(D) Ca^{2+} dynamics when subjects turn forward to contact with the empty prison. Means ±SEM of N=34 events in 4 WT mice and 29 in 5 KO mice.
(E) Change of Ca^{2+} dynamics before and after turning forward events. Means ±SEM of N=34 events in 4 WT mice and 29 in 5 KO mice. Two-way ANOVA: Effect of genotype $F_{(1,61)}=0.1$, $p=0.67$; effect of pre vs post $F_{(1,61)}=0.002$, $p=0.9$, effect of interaction $F_{(1,61)}=0.02$, $p=0.8$.
(F) Ca^{2+} dynamics when subjects turn forward to contact with the empty prison. Means ±SEM of N=22 events in 4 WT mice and 25 in 5 KO mice.
(G) Change of Ca^{2+} dynamics before and after turning forward events. Means ±SEM of N=22 events in 4 WT mice and 25 in 5 KO mice. Two-way ANOVA: Effect of genotype $F_{(1,45)}=0.8$, $p=0.3$; effect of pre vs post $F_{(1,42)}=4.1$, $**p=0.04$, effect of interaction $F_{(1,42)}=5.7$, $*p=0.02$.

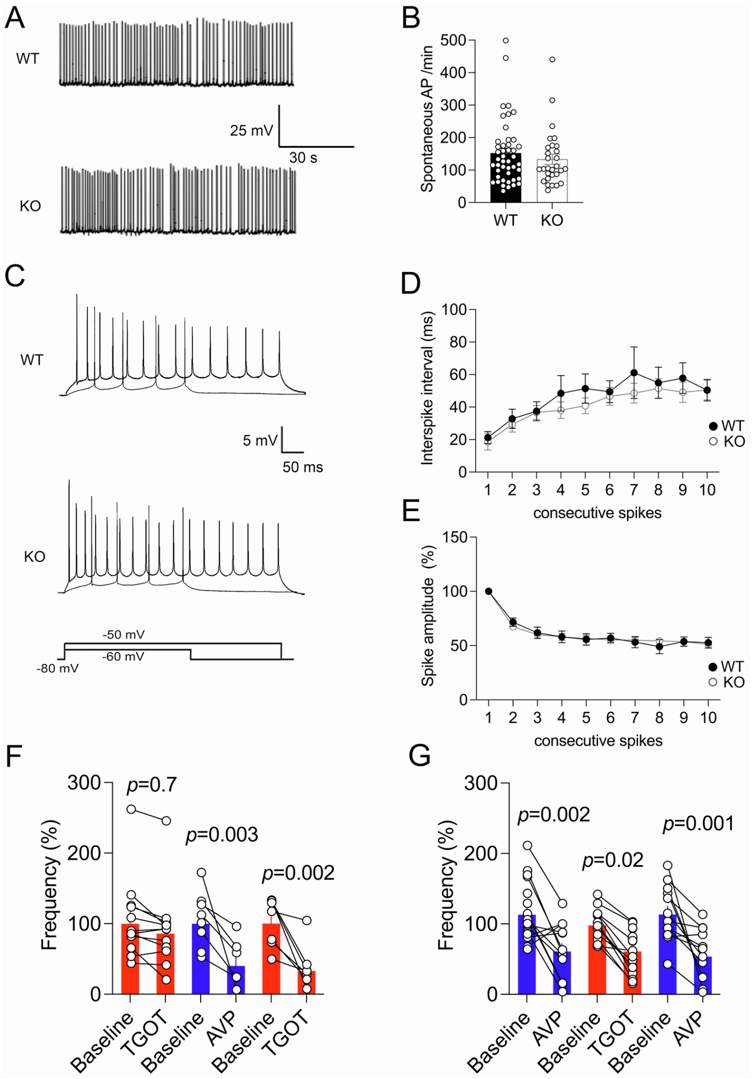

Figure S2. No effect of genotype on the basal firing rate of LS cells ex vivo, and inhibition of SST cells by OXT depends on prior AVP exposure (related to Figure 2).

(A) Patch clamp recording of action potentials in whole cell configuration in acute coronal slices of LS.

(B) The frequency of action potentials is not different between genotypes. Mann-Whitney test p=0.37, n=45 WT cells, 29 KO cells.

(C) Effect of depolarization step on basal firing rate of LS cells.

(D) No effect of genotype on inter-spike intervals. Data are means+/-SEM, n=11 WT and 7 KO cells.

(E) No effect of genotype on spike amplitude. Data are means+/-SEM, n=11 WT and 7 KO cells.

(F) Cells were presented first with TGOT then rinsed for 20 min to go back to baseline before stimulation with AVP then rinsed for 20 min to go back to baseline before stimulation with TGOT. We found no effect of TGOT on the frequency of action potentials when presented first albeit AVP could reduce the firing of these cells as did TGOT after that. Two-way ANOVA indicates an effect of the stimulation with AVP then with TGOT $F_{(1,11)}$=44, p<0.0001 and post-hoc comparisons with Tukey test as indicated.

(G) Cells were presented first with AVP then rinsed for 20 min to go back to baseline before stimulation with TGOT then rinsed for 20 min to go back to baseline before stimulation with AVP. We found that AVP was sufficient to reduce the frequency of action potentials whereas the effect of TGOT depended on prior exposure with AVP. Two-way ANOVA indicates an effect of the stimulation with AVP then with TGOT $F_{(1,18)}$=47, p<0.0001 and post-hoc comparisons with Tukey test as indicated.

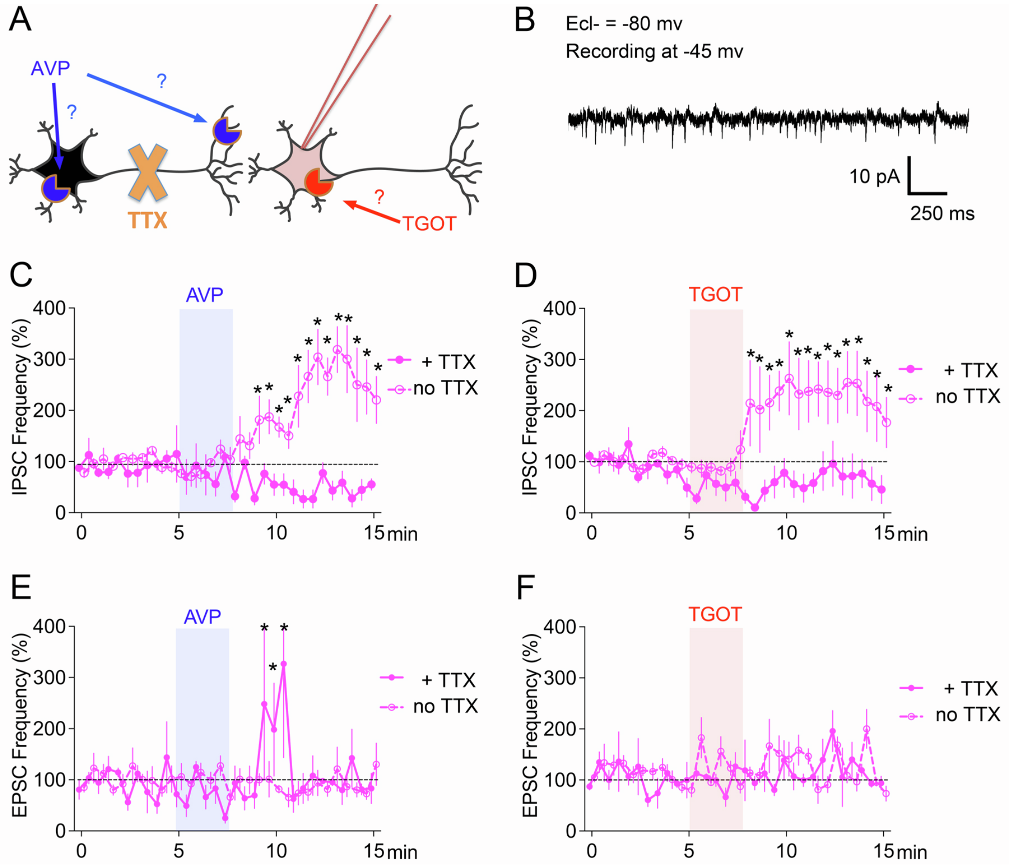

Figure S3. TTX blocked the increase of IPSCs by AVP and TGOT in dLS cells (related to Figure 2).

(A) Where are the putative receptors for AVP and TGOT? Application of the NA^+ channel blocker TTX (0.3 μM) on slices would prevent spontaneous network activity on synaptic transmission and help determine if the effects of AVP and TGOT are in the soma or at the synapse.
(B) To distinguish between the inversion potential of glutamatergic currents $E_{Na/K}$ and GABAergic currents E_{Cl}, we artificially set E_{Cl} at -80 mV, $E_{Na/K}$ at 0 mV, and recorded at -45 mV the frequency of excitatory synaptic currents (EPSC) and inhibitory synaptic currents (IPSC). Changes of synaptic current reflects input activity onto dLS cells inhibited by AVP and TGOT.
(C) AVP increased the frequency of IPSCs. Data are means+/-SEM of N=5 cells without TTX and 4 cells with TTX. Two-way ANOVA: Effect of AVP $F_{(30,208)}=2.2$, $p=0.0005$; interaction with TTX $F_{(30,208)}=5.9$, $p<0.0001$ post-hoc comparison with Tukey test as indicated.
(D) TGOT increased the frequency of IPSCs. Data are means+/-SEM of N=5 cells without TTX and 4 cells with TTX. Two-way ANOVA: Effect of TGOT $F_{(30,208)}=2.2$ $p=0.004$; interaction with TTX $F_{(30,208)}=3.3$, $p<0.0001$ post-hoc comparison with Tukey test as indicated.
(E) AVP had no effect on the frequency of EPSCs except if TTX was co-applied, suggesting cryptic AVP signaling via presynaptic receptors on glutamatergic transmission. Data are means+/-SEM of N=4 cells without TTX and 7 cells with TTX. Two-way ANOVA: Effect of AVP $F_{(30,269)}=0.1$, $p=0.7$; interaction with TTX $F_{(30,269)}=2.4$, $p<0.0001$ post-hoc comparison with Tukey test as indicated.
(F) TGOT had no effect on the frequency of EPSCs. Data are means+/-SEM of N=4 cells without TTX and 7 cells with TTX. Two-way ANOVA: Effect of TGOT $F_{(30,234)}=1.3$ $p=0.1$; interaction with TTX $F_{(30,234)}=0.8$, $p=0.6$.

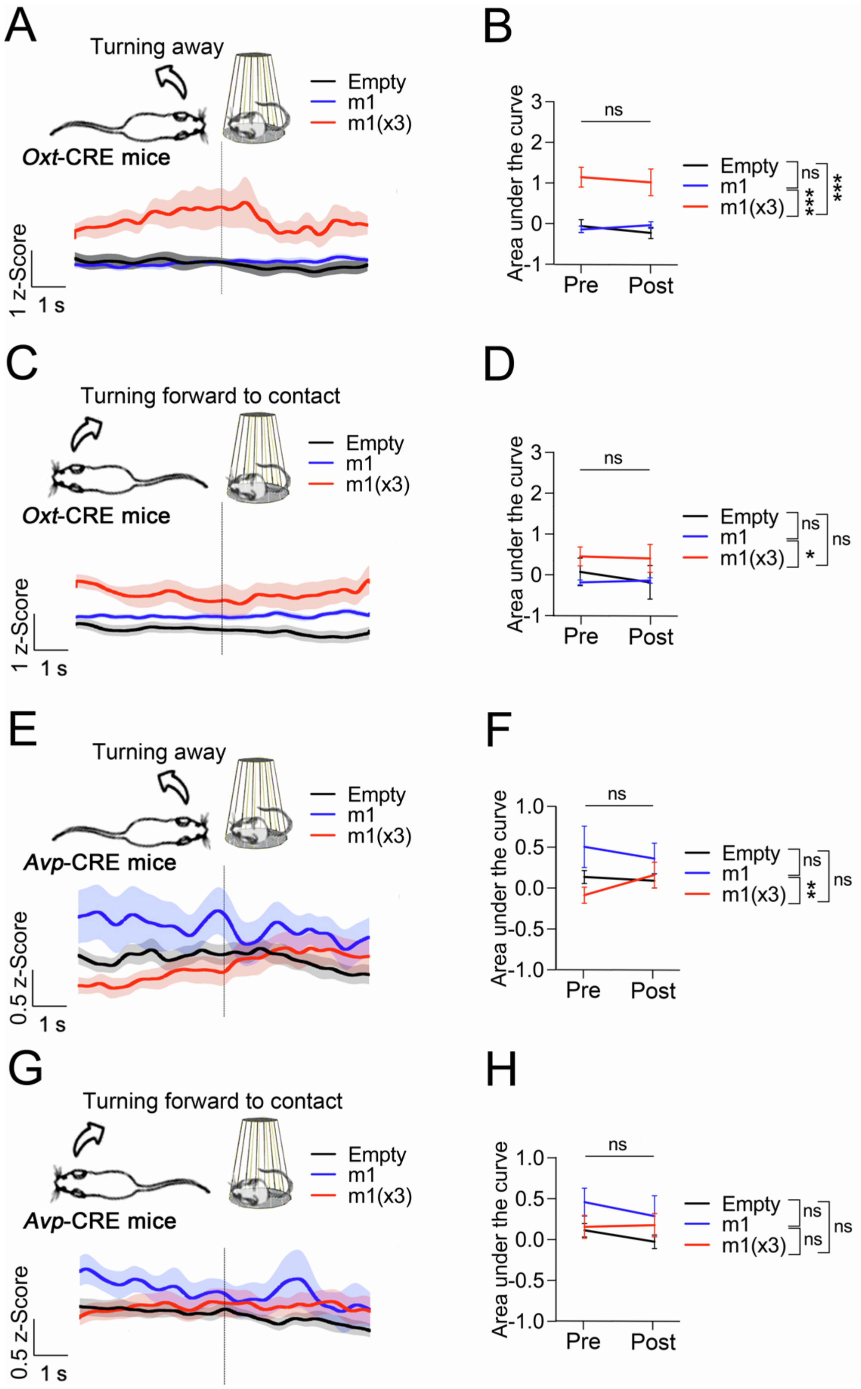

Figure S4. GCaMP7s fluorescence dynamics in the PVN of *Oxt*-CRE mice and *Avp*-CRE mice exploring social and non-social stimuli (related to Figure 4).

(A) Ca^{2+} dynamics in PVN OXT cells during a 10-seconds period surrounding turning away events from the stimulus box either empty or with a mouse. Means ±SEM of N=11 events with empty prison, 22 during social novelty (m1) and 10 during social habituation (m1(x3)). Traces are significantly different in this period: one-way ANOVA $F(2,9639)=102507$, $p<0.0001$, post-hoc Sidak test $p<0.0001$.

(B) Average change of Ca^{2+} dynamics during the 5-seconds period before and 5-seconds period after turning away events. Means ±SEM of N=11 with empty prison, 22 with m1 and 10 with m1(x3) in N=3 *Oxt*-CRE mice. Two-way

ANOVA: Effect of trials $F(2,80)=33.2$, $p<0.0001$; pre versus post $F(1,80)=0.2$, $p=0.6$; interaction $F(2,80)=0.5$, $p=0.5$, post-hoc Tukey test ***$p<0.0001$.
(C) Ca^{2+} dynamics in PVN OXT cells during a 10-seconds period surrounding turning forward to contact with the stimulus box either empty or with a mouse. Means ±SEM of N=11 events with empty prison, 24 during social novelty (m1) and 13 during social habituation (m1(x3)). Traces are significantly different in this period: one-way ANOVA $F(2,9640)=111719$, $p<0.0001$, post-hoc Sidak test $p<0.0001$.
(D) Average change of Ca^{2+} dynamics the 5-seconds period before and 5-seconds period after turning forward to contact events. Means ±SEM of N=11 with empty prison, 24 with m1 and 13 with m1(x3) in N=3 *Oxt*-CRE mice. Two-way ANOVA: Effect of trials $F(2,40)=5.7$, $p=0.006$; pre versus post $F(1,50)=0.2$, $p=0.6$; interaction $F(2,40)=0.28$, $p=0.7$, post-hoc Tukey test *$p=0.03$.
(E) Ca^{2+} dynamics in PVN AVP cells during a 10-seconds period surrounding turning away events from the stimulus box either empty or with a mouse. Means ±SEM of N=11 events with empty prison, 22 during social novelty (m1) and 10 during social habituation (m1(x3)). Traces are significantly different in this period: one-way ANOVA $F(2,9640)=14057$, $p<0.0001$, post-hoc Sidak test $p<0.0001$.
(F) Average change of Ca^{2+} dynamics the 5-seconds period before and the 5-seconds period after turning away events. Means ±SEM of N=38 with empty prison, 21 with m1 and 27 with m1(x3) in N=3 *AVP*-CRE mice. Two-way ANOVA: Effect of trials $F(2,84)=3.8$, $p=0.02$; pre versus post $F(1,82)=0.001$, $p=0.9$; interaction $F(2,84)=1.2$, $p=0.3$, post-hoc Tukey test **$p=0.009$.
(G) Ca^{2+} dynamics in PVN AVP cells during a 10-seconds period surrounding turning forward to contact with the stimulus box either empty or with a mouse. Means ±SEM of N=34 with empty prison, 13 during social novelty (m1) and 28 with m1(x3) during social habituation (m1(x3)). Traces are significantly different in this period: one-way ANOVA $F(2,9640)=15498$, $p<0.0001$, post-hoc Sidak test $p<0.0001$.
(H) Average change of Ca^{2+} dynamics the 5-seconds period before and the 5-seconds period after turning forward to contact events. Means ±SEM of N=34 with empty prison, 13 with m1 and 28 with m1(x3) in N=3 *AVP*-CRE mice. Two-way ANOVA: Effect of trials $F(2,144)=2.4$, $p=0.08$; pre versus post $F(1,144)=0.7$, $p=0.4$; interaction $F(2,144)=0.2$, $p=0.7$.

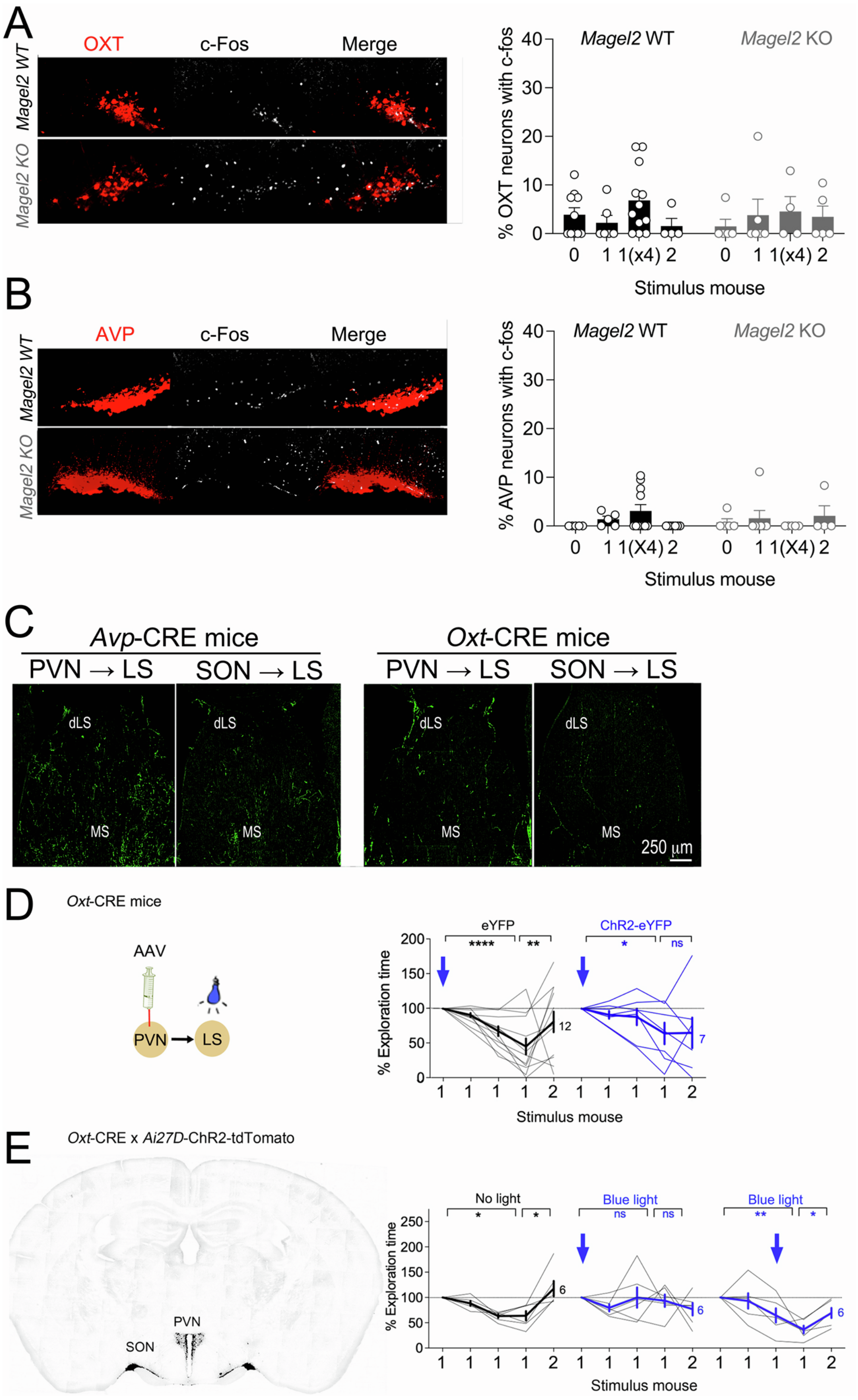

Figure S5. Pathway-specific effects of PVN→LS fibers depend on social interaction phase (novelty versus habituation) (related to Figure 4 and Figure 5).

(A) Immunostaining with NPI-OXT and c-fos antibodies in the SON nuclei. Brains were collected 0.5h after exposure to the known stimulus mouse 4 times. Scale = 150 µm. Proportion of Fos-activated OXT cells engaged in

absence or presence of the stimulus mouse once then 4 times to become familiar. Pairwise comparisons between groups with Mann-Whitney test (means ±SEM in at least 4 mice/group). No effect of stimulation or genotype.

(B) Immunostaining with NPII-AVP and c-fos antibodies in the SON. Scale = 150 µm. Proportion of Fos-activated AVP cells in absence or presence of the stimulus mouse once or 4 times to become familiar. Pairwise comparisons between groups with Mann-Whitney test (means ±SEM in at least 4 mice/group). No effect of stimulation or genotype.

(C) YFP fibers in the LS from hypothalamic OXT and AVP neurons. Avp-CRE and Oxt-CRE mice were injected with an AAV virus DIO-eYFP either in the PVN or in the SON bilaterally. Immunostaining with YFP antibodies shows fibers going all the way up to dLS.

(D) *Oxt*-CRE mice were injected in the PVN with AAV virus DIO-ChR2-eYFP (or eYFP as control) and optic fibers implanted atop dLS bilaterally for optogenetic stimulation during social novelty. Recombination of transgenes was verified in postmortem brain sections with NPI-OXT and YFP antibodies. Infection rate was 64%. Data are means+/-SEM of N=12 eYFP and 7 ChR2 mice. Two-way ANOVA: Effect of ChR2 $F_{(1,18)}=0.01$, $p=0.8$; effect of trials $F_{(4,68)}=8$, $p<0.0001$; interaction $F_{(4,68)}=1.2$, $p=0.2$ post-hoc comparison with Sidak test. Within group comparisons are indicated. Between group comparisons shows no difference.

(E) Crossing *Oxt*-CRE mice with Ai27-ChR2 mice resulted in recombination and expression of ChR2-tdTomato in all OXT neurons including SON and PVN sending fibers in dLS. Recombination of transgene was verified in postmortem brain sections with NPI-OXT and RFP antibodies. Infection rate was 93% in OXT neurons. Mice were implanted with optic fibers atop dLS for optogenetic stimulation during social novelty or during social habituation. Data are means+/-SEM of N=6 mice/group. Two-way ANOVA: Effect of ChR2 stimulations $F_{(2,15)}=2.2$, $p=0.13$; interaction with trial of stimulation $F_{(8,60)}=4$, $p=0.0007$ post-hoc comparison with Dunnett test. Within group comparisons are as indicated. Between group comparisons are as follow for habituation: No light vs blue light at novelty $p=0.03$ and No light vs blue light at habituation $p=0.9$; for discrimination: No light vs blue light at novelty $p=0.016$ and No light vs blue light at habituation $p=0.004$.
